# Bispecific Antibodies in Glioblastoma: Mechanistic Advances, Delivery Innovations, and Translational Challenges in Overcoming Immune Escape

**DOI:** 10.7150/ijms.121829

**Published:** 2025-10-01

**Authors:** He-Lu Wang, Nan Chi, Hong-Tao Zhang

**Affiliations:** 1 School of Clinical Medicine, Shandong Second Medical University, Weifang, China.; 2 Neurosurgery Department of Yantai Yuhuangding Hospital, Yantai, China.; H-L W and N C contributed equally to this work.

**Keywords:** Bispecific Antibodies, Glioblastoma, Blood-Brain Barrier, Tumor Heterogeneity, Immunosuppressive Microenvironment, T-cell Redirection.

## Abstract

The application of bispecific antibody (BsAbs)-based therapeutic strategies for glioblastoma (GBM) has shown considerable promise. By concurrently targeting tumor-associated antigens and immune effector cells, BsAbs can traverse the blood-brain barrier, modulate the immunosuppressive tumor microenvironment, and surmount challenges such as intratumoral heterogeneity and immune evasion. Accumulating evidence indicates that BsAbs surpass conventional monoclonal antibodies and chimeric antigen receptor T cell therapies in the context of GBM through mechanisms that include the redirection of immune cells, blockade of immune checkpoints, and synergistic inhibition of oncogenic signaling pathways. Although constrained by limitations in intracerebral delivery efficiency and the potential for immune-related adverse events, BsAbs represent a promising new frontier in GBM immunotherapy. They particularly enhance therapeutic precision and durability, underscoring their potential as a transformative approach for managing this aggressive malignancy.

## Introduction

Glioblastoma (GBM) is the most aggressive primary brain tumor, with an annual incidence of 3.19 per 100,000 individuals 1. Median overall survival is less than 15 months, and the 5-year survival rate is only 6.8% 2. The current standard of care—maximal safe resection followed by radiotherapy with concomitant and adjuvant temozolomide—affords transient disease control; however, relapse is almost universal 3. Second-line agents, including bevacizumab, offer limited survival benefit 4. The blood-brain barrier (BBB) is a major impediment to therapeutic delivery; coupled with marked intratumoral heterogeneity and dynamic antigen loss, it further undermines treatment efficacy 5, 6. Even the addition of Tumor Treating Fields prolongs survival by only a few months, and overall outcomes remain poor 7.

Despite transformative benefits in other solid tumors, immunotherapies have yielded limited efficacy in GBM 8. Three overarching barriers constrain approaches such as immune checkpoint inhibitors (ICIs), chimeric antigen receptor (CAR) T cells, and oncolytic viruses (OVs): (1) restricted delivery across the BBB; (2) a profoundly immunosuppressive tumor microenvironment (TME) that fosters T-cell dysfunction and exhaustion; and (3) pervasive intratumoral heterogeneity that enables antigen escape 9-11. ICIs, for example, are thwarted by GBM's “cold” phenotype—scarce effector T-cell infiltration within a suppressive TME 12. Although CAR T cells are compelling preclinically, translation is limited by individualized manufacturing, on-target/off-tumor toxicities (e.g., with epidermal growth factor receptor (EGFR)-directed products), and premature T-cell exhaustion 13. Antigen loss variants and TME-driven upregulation of inhibitory checkpoints further diminish activity, emphasizing the dual need to address heterogeneity and reverse immunosuppression 14.

**Figure 1:**
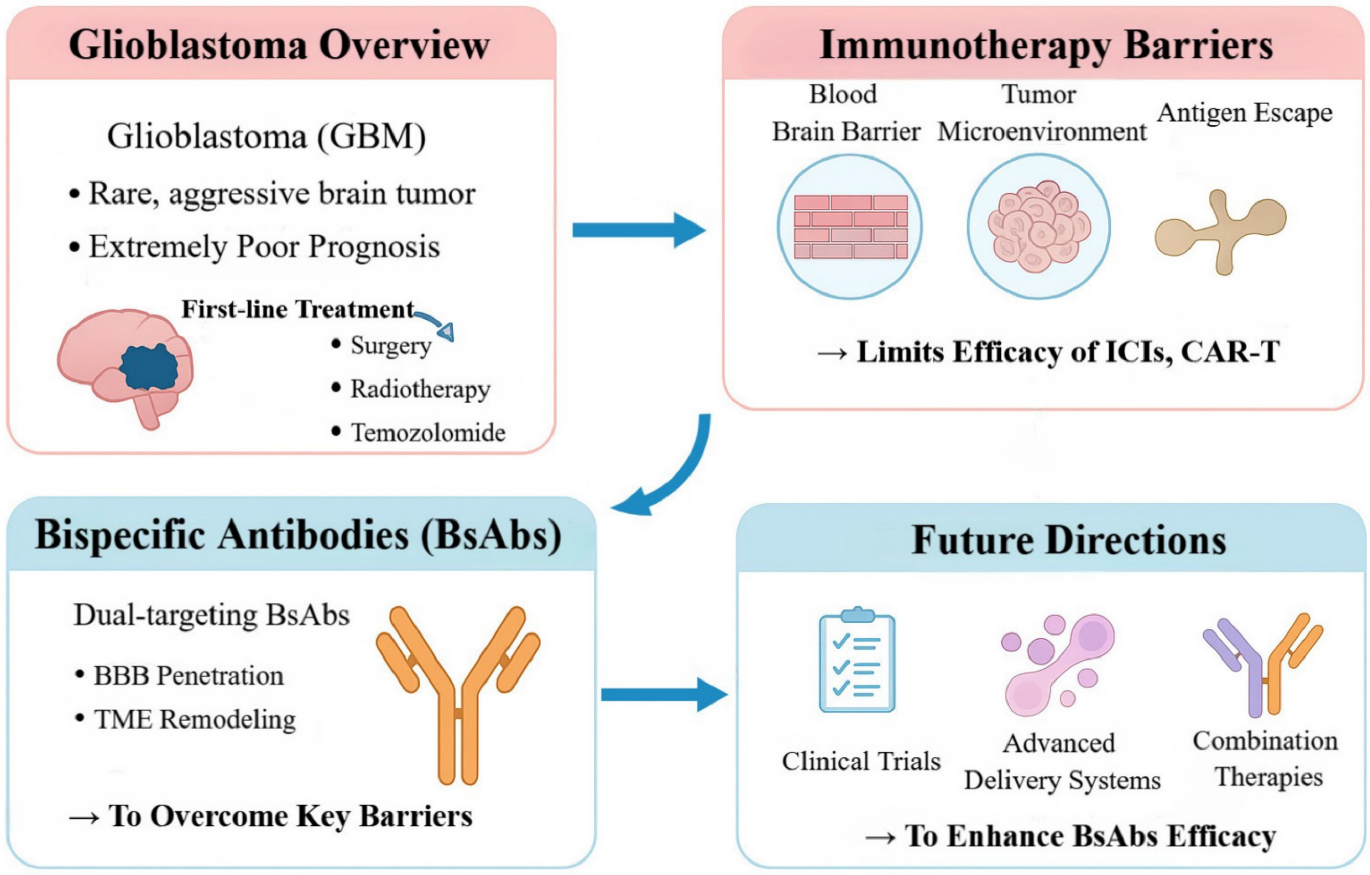
** Current Challenges and BsAbs Strategies in Glioblastoma Immunotherapy.** This figure outlines the key obstacles to immunotherapy in GBM—including the blood-brain barrier, immunosuppressive microenvironment, and antigen escape—and how BsAbs overcome these limitations. By enabling brain penetration and reprogramming the tumor immune milieu, BsAbs offer a promising therapeutic avenue. Future advancements will rely on improved delivery systems and combination therapies to enhance efficacy.

Locally immune-activating approaches (for example, the oncolytic herpesvirus G47Δ) broaden therapeutic options, but their ability to effect durable, systemic immune reprogramming is limited. Consequently, modalities are needed that traverse physiological barriers, precisely target malignant cells, and re-engineer antitumor immunity. Bispecific antibodies (BsAbs)—readily deployable “off-the-shelf” agents capable of precise immune modulation—have emerged as a promising strategy to circumvent entrenched bottlenecks in GBM (Figure 1) 15. By engaging two targets within a single molecule, BsAbs can redirect T and/or NK cells, remodel the TME, and exploit cooperative co-targeting mechanisms 16. Relative to CAR T-cell therapy, BsAbs obviate bespoke manufacturing and may reduce cost; relative to conventional monoclonal antibodies, their dual mechanisms can more effectively disrupt immunosuppressive circuits 17. Their architectural flexibility confers further advantages: enabling receptor-mediated transport across the BBB to enhance central nervous system (CNS) delivery; dual-antigen recognition to limit antigen loss; and either lymphocyte redirection or blockade of inhibitory axes (e.g. CD47/signal regulatory protein alpha) to convert a “cold” TME into an inflamed, effector-permissive state 18.

In this Review, we delineate mechanisms of action, target pairings, and delivery paradigms for BsAbs in GBM; critically evaluate advances from preclinical studies and clinical trials; and examine the translational promise and limitations of next-generation delivery platforms, including stimulus-responsive nanocarriers. We also define priorities for optimizing CNS delivery, mitigating toxicities (e.g., cytokine-release syndrome(CRS)), engineering dual-antigen constructs to counter heterogeneity and antigen escape, and designing rational combination regimens. By integrating cross-disciplinary progress with unmet clinical needs, we provide a framework to guide the rational development of BsAbs and to inform individualized immunotherapeutic strategies for GBM.

## Mechanistic Basis of BsAb-Mediated Tumor-Immune Cell Bridging

Against this backdrop, a rigorous understanding of the molecular mechanisms and structural classes of BsAbs provides the conceptual foundation for advancing clinical translation. BsAbs are genetically engineered immunotherapeutics whose cardinal advantage is their capacity to engage two distinct epitopes with a single molecule, thereby overcoming limitations inherent to conventional monoclonal antibodies 19, 20. Structurally, BsAbs are broadly classified into two architectures. (1) Non-IgG-like, short-chain formats (for example, bispecific T-cell engagers (BiTEs)) consist of two single-chain variable fragments (scFvs) linked by flexible peptide spacers; these ~55-kDa molecules exhibit rapid extravasation and deep tissue penetration. (2) IgG-like, full-length formats (~150 kDa) retain an Fc domain and can mediate antibody-dependent cellular cytotoxicity (ADCC) and complement-dependent cytotoxicity (CDC) 21, 22. However, their tissue penetration is comparatively limited, and Fcγ-receptor (FcγR)-mediated off-target activation may precipitate CRS. To balance efficacy and safety, optimization strategies include Fc-silencing mutations (for example, L234A/L235A or L234F/L235E) or selection of low-effector IgG subclasses (for example, IgG4) 23, 24.

Mechanistically, BsAbs exert antitumor activity through three principal paradigms that leverage spatial “bridging.” First, effector-cell redirection: BsAbs can simultaneously bind receptors on immune cells (such as cluster of differentiation 3ε (CD3ε) on T cells or CD16 on natural killer (NK) cells) and tumor-associated antigens (TAAs), thereby promoting immunological-synapse formation, bypassing major histocompatibility complex (MHC) class I-restricted antigen recognition, and directly activating effector cells to proliferate and release perforin and granzyme B (GZMB) to kill tumor cells—an especially valuable property in GBM with its “cold” phenotype 25, 26. BiTEs exemplify this mechanism: their compact architecture favors tumor penetration and they indiscriminately engage CD3⁺ T-cell subsets—including CD4⁺, CD8⁺, and Tregs—thereby circumventing native T-cell receptor (TCR) specificity. Their clinical deployment, however, is constrained by short serum half-lives necessitating continuous infusion and by the risk of CRS 27.

**Figure 2:**
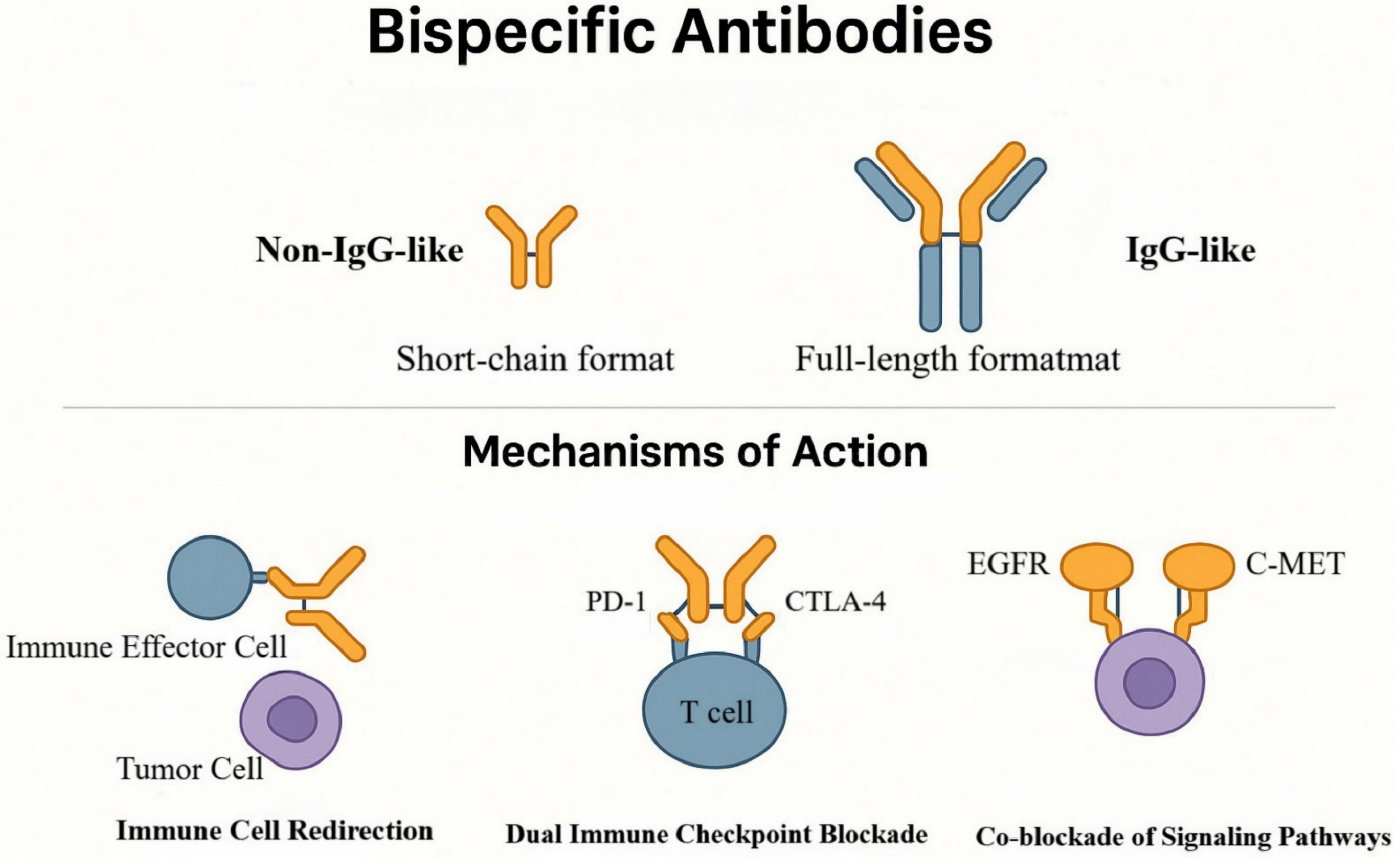
** Structural Classes and Mechanisms of BsAbs.** This figure summarizes BsAbs' key structural types and three bridging-based mechanisms. As single molecules binding two distinct epitopes, BsAbs comprise Fc-less non-IgG-like formats and full-length IgG-like structures. Their antitumor effects include immune cell redirection, dual checkpoint blockade (e.g., PD-1/CTLA-4), and co-blockade of signaling pathways (e.g., EGFR/c-MET).

Second, dual checkpoint blockade: BsAbs can concurrently inhibit two inhibitory immune checkpoints (for example, programmed cell death protein 1 (PD-1)/cytotoxic T-lymphocyte-associated protein 4 (CTLA-4) or PD-1/T-cell immunoglobulin and mucin-domain containing-3 (TIM-3)), thereby synergistically releasing immunosuppressive signaling within the TME and augmenting T-cell-mediated antitumor responses 28. Given the frequent co-expression of multiple suppressive axes in GBM, dual checkpoint blockade may achieve deeper and more durable reinvigoration. Third, coordinated pathway blockade: BsAbs can co-target two oncogenic receptors or ligands on tumor cells; by disrupting receptor dimerization and parallel signaling, they attenuate survival pathways and forestall resistance—an approach particularly pertinent to GBM subtypes characterized by signaling redundancy or therapy resistance (Figure 2) 29.

## Translational Lessons from Blood Cancers: Optimizing BsAbs for Solid Tumors

Clinical successes of BsAbs in hematologic malignancies have validated the modality and provided a blueprint for translation to solid tumors, including GBM 30. Blinatumomab (CD19×CD3) exemplifies this paradigm: by bridging T cells and tumor cells to form cytolytic immunological synapses, it improves outcomes in relapsed or refractory B-cell acute lymphoblastic leukemia and demonstrates superiority in clearing minimal residual disease, thereby substantiating the clinical feasibility of T-cell redirection 31. Additional BsAbs targeting CD20×CD3 (e.g., glofitamab) and B-cell maturation antigen ×CD3 (e.g., teclistamab) have shown robust activity in lymphoma and multiple myeloma, further underscoring the breadth of this platform across hematologic cancers 32, 33. From these experiences, several design principles have emerged: affinity tuning to enhance safety; rigorous selection of highly specific or lineage-restricted antigens to improve targeting precision; and dose- and schedule-optimization to sustain therapeutic exposure.

The development of trispecific antibodies (TsAbs) in hematologic malignancies has extended multi-target strategies by enabling a single molecule to engage multiple antigens or receptors, thereby augmenting potency and curbing antigen escape. The resulting principles—affinity modulation, target-selection criteria, dosing optimization, and multi-target architecture—directly inform BsAb design for GBM. This concept is already being applied in GBM through constructs such as dual-tumor-antigen trispecific T-cell engagers, which simultaneously bind two TAAs and CD3 to mitigate heterogeneity-driven antigen loss; preclinical studies report greater antitumor activity than bispecific counterparts 34.

Notwithstanding these advances, the pathobiology of solid tumors imposes additional constraints on BsAbs, including limited drug distribution across the BBB, pronounced intratumoral heterogeneity, and a profoundly immunosuppressive TME enriched in transforming growth factor-β (TGF-β) and tumor-associated macrophages (TAMs). Aligned with lessons from hematologic malignancies and tailored to GBM-specific hurdles, current BsAb efforts in GBM coalesce around three translational thrusts: (1) adapting T-cell redirection via CD3- or CD16-engaging BsAbs against GBM-relevant antigens such as epidermal growth factor receptor vIII (EGFRvIII), interleukin-13 receptor subunit alpha-2 (IL-13Rα2), and human epidermal growth factor receptor 2 (HER2) 35; (2) recalibrating the immune milieu through dual checkpoint blockade (e.g., PD-1/CTLA-4) or "antigen + immunomodulator" designs (e.g., co-targeting a TAA and TGF-β) 36; and (3) innovating delivery paradigms to surmount the BBB—a central prerequisite for breaking current therapeutic bottlenecks 37.

## BsAbs Target Combinations Addressing Core Challenges of GBM

### Ang-2/TSPO Co-blockade: Reversing Anti-angiogenic Resistance

Informed by advances in hematologic malignancies, the rational design of GBM-specific target combinations that reflect its distinctive pathobiology and immune milieu has become a central focus for BsAb development. GBM is highly vascular and profoundly immunosuppressive, and it rapidly acquires complex adaptive resistance to anti-angiogenic therapy 38. Among the mediators implicated in this process, angiopoietin-2 (Ang-2) and the 18-kDa translocator protein (TSPO) have emerged as priority targets for combinatorial strategies 39. Ang-2 is a key pro-angiogenic cue that orchestrates vessel initiation, remodeling, and sprouting, and its expression is markedly upregulated following bevacizumab exposure 40. TSPO, an outer-mitochondrial-membrane protein widely overexpressed in GBM, enhances resistance to chemotherapy and radiotherapy by restraining the caspase-9/Apaf-1-dependent mitochondrial apoptosis pathway 41. In bevacizumab-treated GBM, Ang-2 and TSPO show pronounced co-expression and spatial colocalization within tumor-derived endothelium, establishing a dual resistance barrier 42. Ang-2 drives pathological angiogenesis, vascular leak, and recruitment of immunosuppressive cells such as TAMs 40, 43, whereas high TSPO expression amplifies anti-apoptotic signaling that protects tumor cells from therapy-induced death 44. Collectively, these mechanisms rationalize failures of anti-angiogenic therapy and motivate interventions that concurrently modulate angiogenesis and apoptosis.

**Figure 3:**
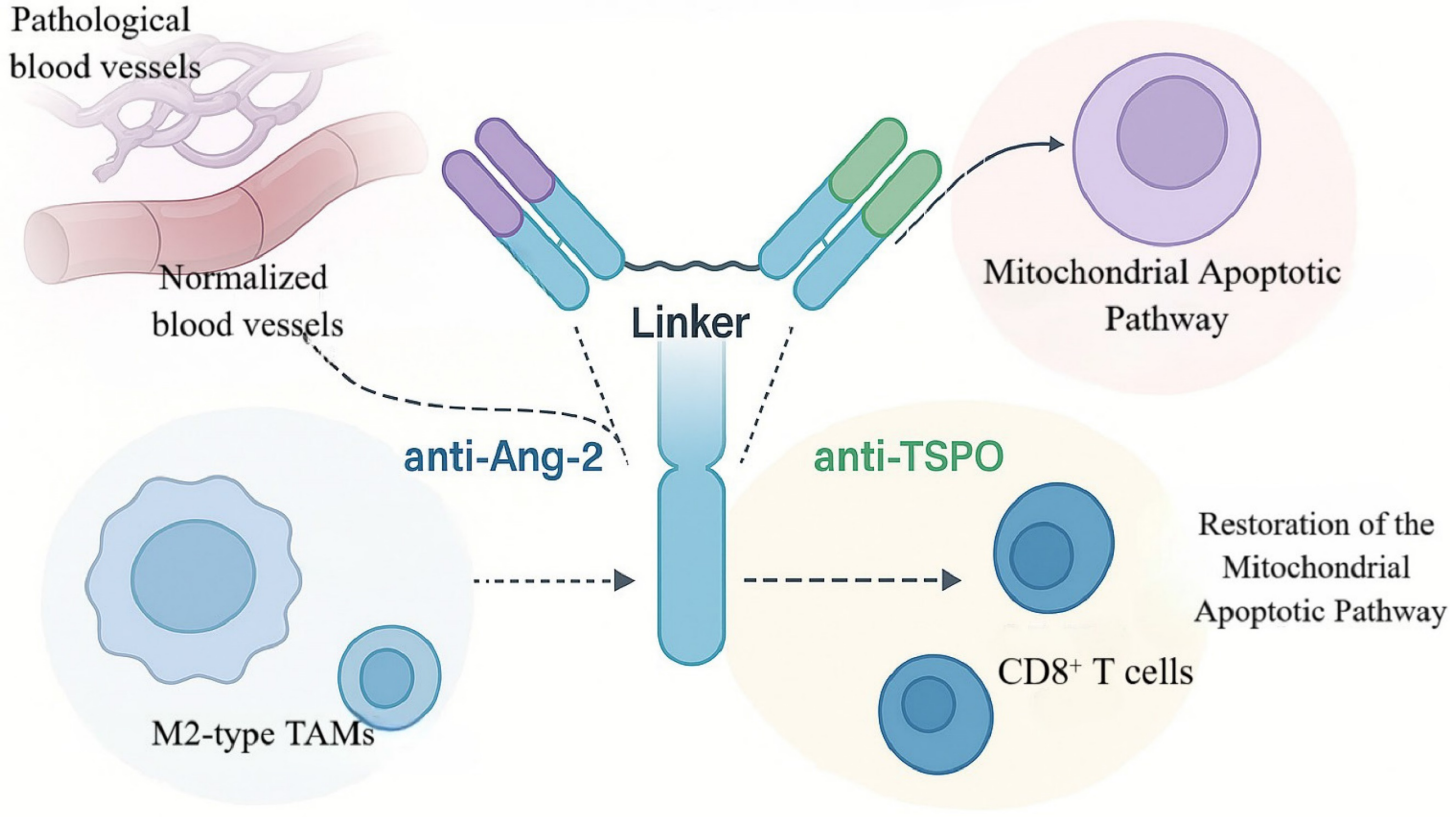
** Synergistic therapeutic mechanism of the bispecific antibody ScBsAbsAng-2/TSPO.** The schematic illustrates how ScBsAbsAng-2/TSPO simultaneously blocks Ang-2 and TSPO to synergistically treat GBM: its anti-angiogenic domain promotes normalization of pathological vasculature, while the pro-apoptotic domain restores mitochondrial apoptotic function. Together, they induce remodeling of the immune microenvironment characterized by increased CD8⁺ T cell infiltration and reduced M2-type TAMs.

On this pathophysiological basis, Zhou and colleagues engineered a bispecific antibody, ScBsAbsAng-2/TSPO, to simultaneously blunt Ang-2-driven vascular abnormalities and TSPO-mediated apoptotic escape 45. The molecule adopts an scFv-based architecture in which anti-Ang-2 and anti-TSPO domains are linked by a flexible peptide spacer to achieve coordinated, intratumoral co-targeting within a single construct 46. Functionally, the Ang-2-binding arm suppressed aberrant neovascularization, reduced TAM infiltration by ~40%, and decreased expression of pro-angiogenic factors, whereas the TSPO-binding arm restored mitochondrial apoptotic signaling with a significant increase in caspase-9 activation (Figure 3) 45, 47.

In the C6 glioma rat model, ScBsAbsAng-2/TSPO reduced endothelial co-expression of Ang-2/TSPO and increased tumor-cell apoptosis by 3.2-fold. Median survival extended to 72 days versus 44 days in controls—a 63.6% improvement—surpassing monoclonal or single-target comparators. The antibody also reshaped the TME: M2-polarized TAMs decreased by 35%, whereas intratumoral CD8⁺ T-cell density increased 1.8-fold, indicating ancillary immunomodulatory effects. Notwithstanding these gains, ~60% of treated animals survived with residual tumor, and immunosuppressive TAM subsets persisted, underscoring the difficulty of fully reprogramming the microenvironment 48.

Across *in vitro* assays and orthotopic brain-tumor models, ScBsAbsAng-2/TSPO showed favorable antitumor activity with an acceptable safety profile, supporting its candidacy as an immunotherapeutic agent. This approach combines structural simplicity with an expanded therapeutic window and integrated microenvironmental modulation; however, its efficiency in traversing the BBB and its capacity to elicit durable responses require further comparative validation 45.

### A2V Antibody: Dual Anti-angiogenesis with TAM Reprogramming

**Figure 4:**
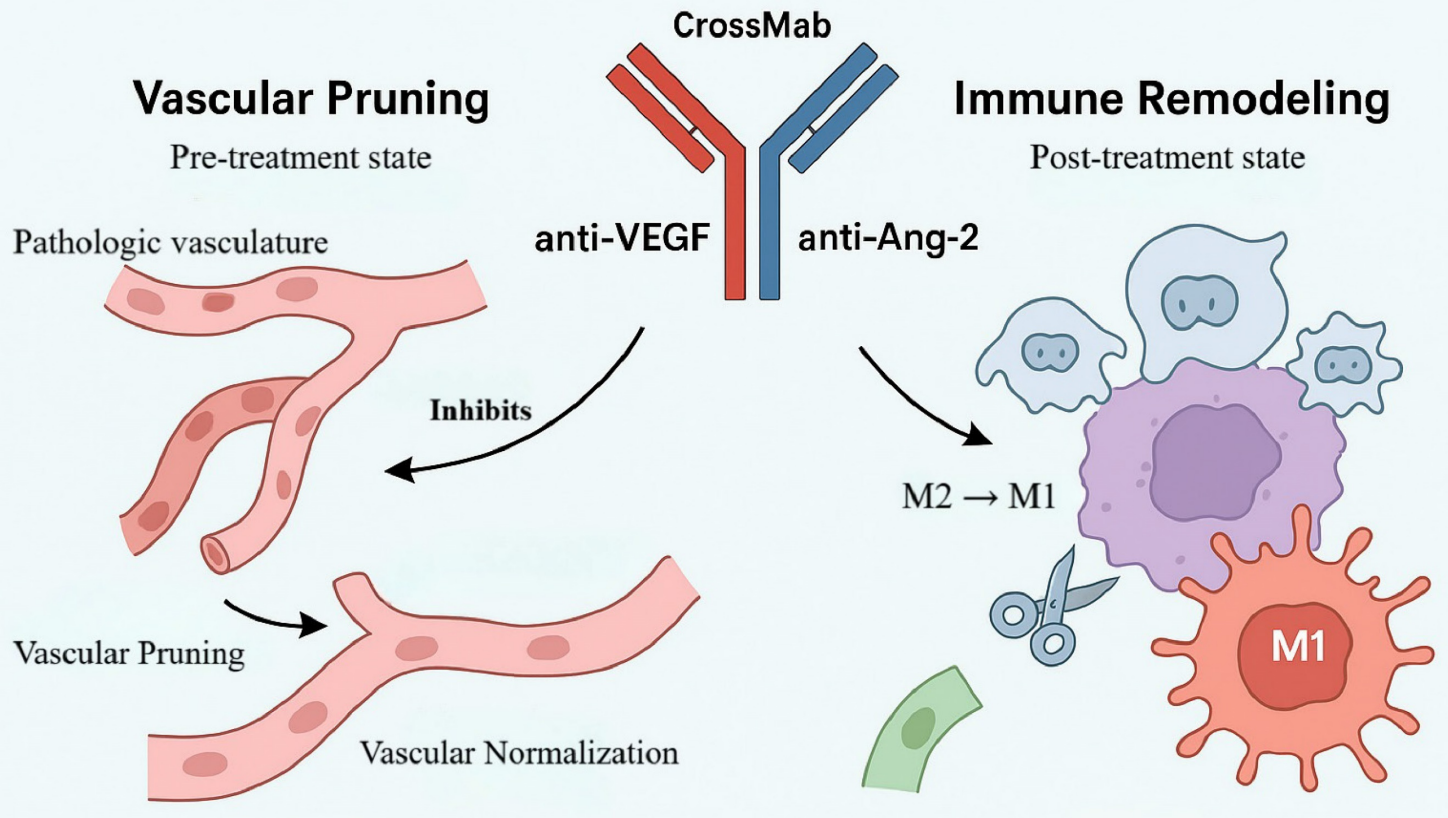
** Mechanism of A2V-mediated dual targeting of VEGF/Ang-2 in vascular remodeling and immune microenvironment regulation.** This schematic illustrates the dual mechanisms of the bispecific antibody A2V in glioma treatment through coordinated targeting of VEGF and Ang-2. It eliminates abnormal vasculature via "vascular pruning" while promoting vascular normalization, and concurrently drives macrophage polarization from pro-tumorigenic M2 to anti-tumorigenic M1 phenotypes. This immune remodeling ultimately overcomes drug resistance and suppresses tumor progression.

Beyond modulating apoptotic and angiogenic pathways, concomitant inhibition of multiple angiogenic mediators is an effective means of overcoming resistance to anti-angiogenic therapy. In GBM, dysregulated angiogenesis sustains tumor growth and invasiveness 49. Vascular endothelial growth factor (VEGF) is a principal pro-angiogenic driver that promotes endothelial proliferation and migration and fuels neovascularization, thereby maintaining a continuous supply of nutrients and oxygen to the tumor. Consequently, VEGF has become the primary target of anti-angiogenic strategies (for example, bevacizumab) 50. However, VEGF blockade alone is often accompanied by compensatory upregulation of Ang-2. Ang-2 regulates vascular stability and triggers endothelial sprouting; under VEGF inhibition it promotes vessel destabilization, exacerbates aberrant vascular architecture, and polarizes TAMs toward a pro-angiogenic M2 phenotype, collectively driving resistance 51. Thus, VEGF and Ang-2 operate as a complementary, cooperative axis within the GBM microenvironment. Single-target intervention is unlikely to disrupt this positive feedback, whereas dual blockade affords greater leverage to intercept the pathological network (Figure 4).

On this rationale, BsAbs that co-target VEGF and Ang-2 represent a next-generation anti-angiogenic approach. A CrossMab-based bispecific antibody, A2V, simultaneously inhibits VEGF- and Ang-2-dependent signaling to extinguish aberrant vessel formation and interrupt resistance feedback at its source, thereby achieving more comprehensive control of the tumor's "vascular ecology" 52. This concept has been clinically validated in ophthalmology: faricimab (a VEGF-A/Ang-2 BsAb) is approved for diabetic macular edema and neovascular age-related macular degeneration and has demonstrated efficacy and safety advantages over monoclonal comparators 53. In addition, domestically developed candidates IBI324 and ASKG712 have entered phase I clinical trials, indicating platform maturity and feasibility of this target combination and providing a practical foundation for extension to solid tumors such as GBM.

In the syngeneic GL261 murine glioma model, A2V outperformed the anti-VEGF monoclonal antibody B20 in suppressing microvessel formation, significantly reducing intratumoral microvessel density. Notably, A2V selectively pruned immature, pericyte-poor vessels while enhancing pericyte ensheathment of mature vessels—aligning with the therapeutic goal of eliminating pathological vessels while preserving functional ones 48. Mechanistically, A2V dually neutralized endothelial sprouting elicited by combined VEGF and Ang-2 stimulation, whereas B20 only partially inhibited this process, supporting the advantage of dual-target blockade in controlling aberrant revascularization. Moreover, pericyte-rich mature vessels exhibited relative treatment refractoriness, consistent with the hypothesis that dense pericyte coverage shields vessels from anti-angiogenic effects 54.

In a human GBM xenograft model (MGG8), A2V did not substantially alter vascular morphometric parameters yet significantly prolonged survival through mechanisms independent of overt vascular remodeling, suggesting antitumor activity beyond vascular endpoints. Further analyses showed that A2V reprograms the TME by shifting TAM polarization from protumor M2 toward antitumor M1—with expansion of M1 subsets, reduction of M2 subsets, and a net increase in the M1/M2 ratio—without requiring T-cell participation and involving both circulating monocyte-derived macrophages and resident microglia 48. Accordingly, in "vascularly abnormal" GBM, A2V acts primarily via vascular pruning and normalization, whereas in "vascularly mature" GBM its efficacy relies more on immune-microenvironment reprogramming, revealing ecology-dependent mechanistic differences 55.

Collectively, these data indicate that intratumoral vascular phenotypes critically shape therapeutic responses. A2V displays phenotype dependence: in vascular-abnormality-dominant tumors, it restrains progression by pruning immature vessels and promoting vascular normalization; in vascular-mature tumors, it exerts antitumor effects via immune reprogramming. These mechanistic distinctions underscore GBM heterogeneity and support the feasibility of vascular-ecology-informed precision stratification. By repolarizing TAMs toward M1, A2V forges a mechanistic bridge between vascular targeting and immunomodulation, providing a rationale for combinations with ICIs 48. Preliminary phase I clinical data (NCT01688206) suggest a favorable safety profile across multiple solid tumors without severe neurotoxicity or CRS, indicating a potential safety window for CNS applications. Evidence of antitumor activity in GBM further supports extension from ophthalmic indications to highly aggressive tumors, strengthening the translational promise of this dual-target antibody in GBM.

### B7-H3×CD3 Engager: PET-Guided Tumor Targeting

**Figure 5:**
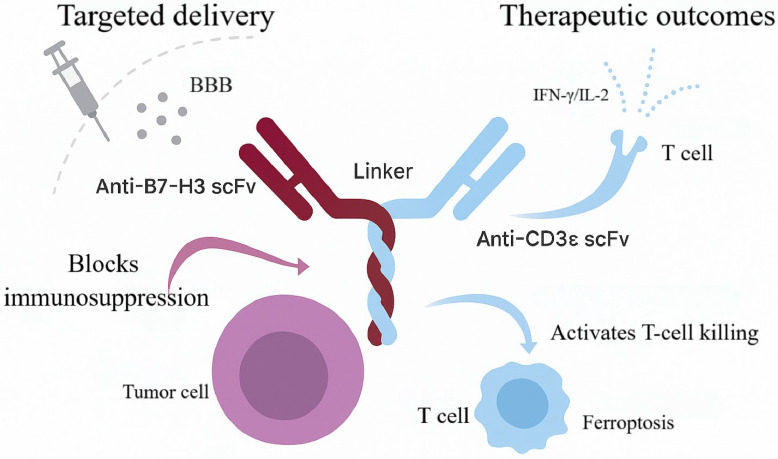
** Schematic diagram of the bispecific antibody-based nanosystem for targeted induction of ferroptosis in brain tumors.** This illustration reveals the key anti-tumor mechanism by which the B7-H3/CD3-targeting nanosystem reverses immunosuppression and activates ferroptosis. Utilizing a smart linker, it crosses the blood-brain barrier, simultaneously engages both tumor and T cells, alleviates immunosuppression, and activates T cells to secrete IFN-γ and IL-2, ultimately leading to the synergistic induction of ferroptosis in tumor cells.

Beyond tumor- and vasculature-directed strategies, directly activating T cells while simultaneously relieving checkpoint-mediated inhibition represents a complementary avenue to amplify antitumor immunity. The immune checkpoint molecule B7 homolog 3 (B7-H3, CD276) functions predominantly as an inhibitory regulator of T-cell-mediated antitumor responses and is highly, selectively overexpressed in most GBM, where expression correlates with grade and poor prognosis 56. The CD3 complex—particularly the ε chain—is the core signal-transducing module of the TCR and is indispensable for antigen-recognition-coupled activation 57. Within the TME, B7-H3 suppresses T-cell activity and facilitates immune evasion, whereas engagement of CD3ε can override this inhibition and restore effector function (Figure 5) 58, 59. Together, B7-H3 and CD3ε delineate an inhibitory-activating axis that provides a clear rationale for BsAbs co-targeting both to relieve immunosuppression, reinvigorate T cells, and promote tumor clearance.

B7-H3×CD3 BsAbs operationalize this concept; among them, MGD009 is a prototypical candidate engineered for high-affinity binding to both B7-H3 and CD3ε, thereby promoting immunological synapse formation between T cells and tumor cells. Surface plasmon resonance (SPR) measured equilibrium dissociation constants (Kd) of 37.4 nM for B7-H3 and 16.1 nM for CD3ε, indicating strong binding with relatively rapid dissociation. Flow cytometry and immunofluorescence further confirmed specific membrane binding of MGD009 to B7-H3-high U-87MG cells, with negligible signal in B7-H3-knockout cells 60. By bridging tumor cells and T cells, MGD009 activates T cells and induces secretion of pro-inflammatory cytokines, including interferon-γ (IFN-γ) and interleukin-2 (IL-2) 59, 61. In preclinical pharmacokinetic studies, ^89Zr-DFO-labeled MGD009 achieved peak tumor uptake of 10.77 ± 1.43% injected dose per gram (%ID/g) at 24 h in a subcutaneous U-87MG model; in an orthotopic brain-tumor model, intrathecal administration further increased tumor uptake to 18.10 ± 0.87%ID/g, with a tumor-to-brain ratio of 14.23 at 24 h—significantly higher than blockade and isotype-control groups (p < 0.05). Major off-target uptake occurred in liver (39.27-41.27%ID/g) and spleen, without significant hematologic toxicity or organ injury 60.

Conventional BsAbs in the CNS are often limited by inefficient BBB penetration and systemic toxicities (e.g., CRS). To mitigate these constraints, an matrix metalloproteinase-2 (MMP-2)-responsive nanodelivery platform (S-biAb/dEGCG@NPs) was developed 62. Leveraging hyaluronic-acid (HA)-mediated CD44 targeting, this system enhances active accumulation at brain-tumor sites. Relative to intravenous dosing, intrathecal administration increased tumor uptake to 18.1%ID/g in an orthotopic GBM model—more than threefold higher than conventional delivery—and thereby improved intratumoral bioavailability 63.

Mechanistically, S-biAb/dEGCG@NPs not only activates T-cell function but also co-modulates solute-transport and ferroptosis pathways 64. By down-regulating glutathione peroxidase 4 (GPX4), the platform synergizes with IFN-γ-induced ferroptotic signaling to intensify tumor-cell lethality, establishing a positive-feedback loop between immune activation and regulated cell death that amplifies antitumor efficacy. In orthotopic GBM mouse models, treatment significantly prolonged survival and was accompanied by increased CD8⁺ T-cell infiltration and reductions in immunosuppressive cellular subsets within the TME. Up-regulation of IFN-γ and IL-2 further supports T-cell-mediated immunity as the principal therapeutic driver 65.

Beyond nanodelivery, MGD009 as monotherapy has advanced into multiple clinical studies, including phase I/II trials (e.g., NCT02952259) evaluating efficacy and safety in patients with GBM. Early findings indicate a manageable toxicity profile without severe immune-related adverse events and favorable CNS tolerability. Positron-emission tomography (PET) imaging demonstrates rapid enrichment and sustained intratumoral retention, consistent with strong tumor targeting and *in vivo* stability. Comparative evaluations of intravenous versus intraventricular administration suggest that intraventricular dosing further enhances drug accumulation and the magnitude of immune activation, informing optimization of BsAb delivery strategies 60.

### EGFRvIII BiTE: Tackling Antigenic Heterogeneity

**Figure 6:**
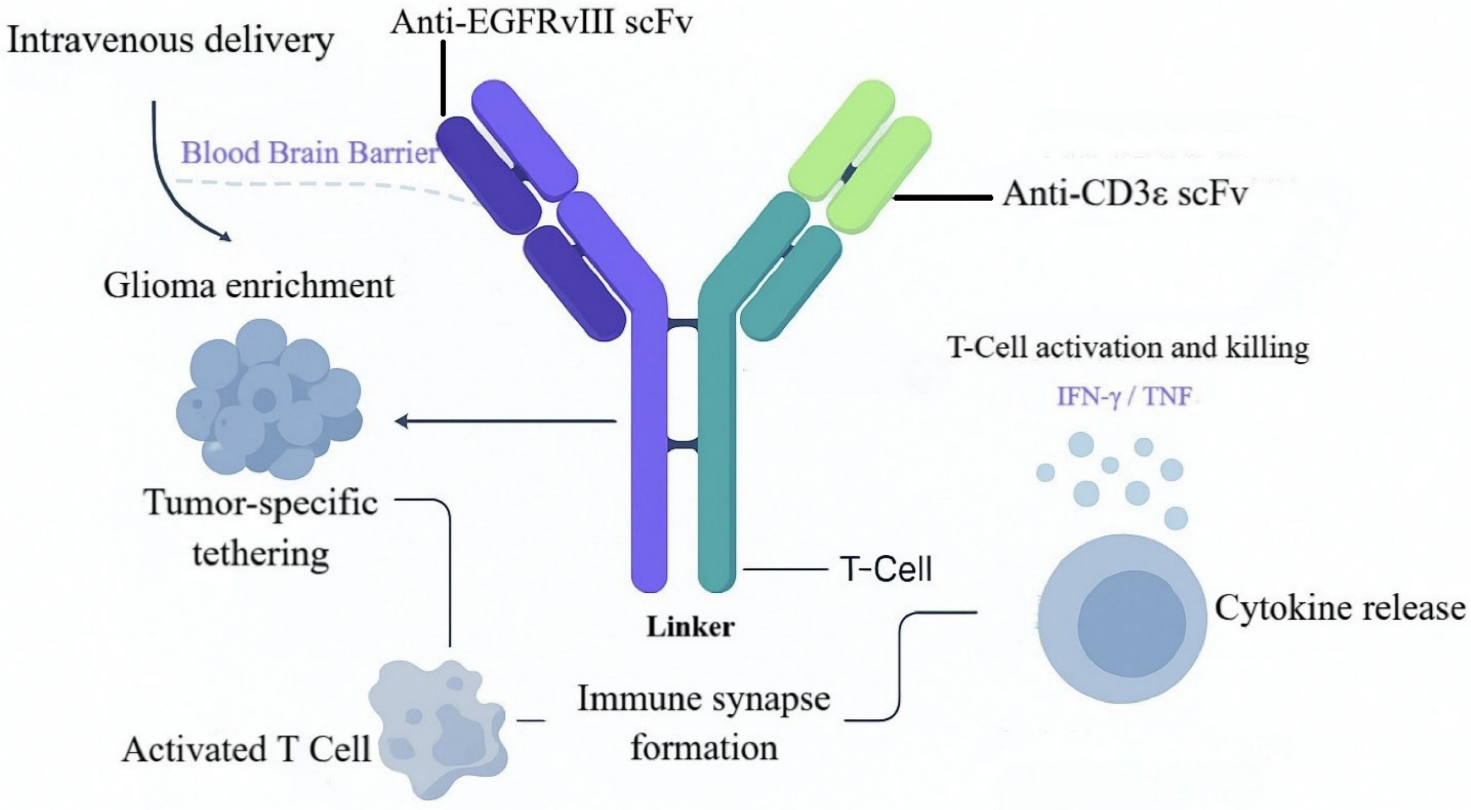
** Mechanism of action of the EGFRvIII-CD3 bispecific antibody.** This schematic illustrates the process of EGFRvIII-CD3 bispecific antibody-mediated targeted therapy for glioma. After intravenous administration, the antibody crosses the blood-brain barrier, enriches at the tumor site, and simultaneously engages both tumor cells and T cells through its dual-targeting properties. This leads to the formation of an immunological synapse, effectively activating T cell-mediated cytotoxicity and promoting the release of cytokines such as IFN-γ and TNF, ultimately resulting in specific elimination of EGFRvIII-positive tumor cells.

Beyond the B7-H3×CD3 paradigm, T-cell-redirecting BsAbs against additional GBM-relevant antigens show substantial promise, with EGFRvIII as a leading target. EGFRvIII is a highly tumor-specific antigen generated by deletion of exons 2-7 in the EGFR gene; it is absent from normal tissues yet stably expressed in ~30% of GBM cases 66. EGFRvIII is constitutively active and drives tumor-cell proliferation, invasion, and therapeutic resistance 67. The CD3 complex is the core signal-transducing module of the TCR, mediating antigen recognition and triggering T-cell activation 68. In GBM, EGFRvIII confers tumor specificity, whereas CD3ε provides T-cell cytolytic potential. Together, these epitopes establish a recognition-effector axis, positioning EGFRvIII×CD3 BsAbs as a strategy to retarget T cells to EGFRvIII-positive GBM and to blunt antigen escape and intratumoral heterogeneity (Figure 6).

In early work, Patrick et al. engineered a fully human EGFRvIII×CD3 BsAb (hEGFRvIII-CD3 bi-scFv), achieving balanced structural stability and functional cooperativity by optimizing variable-domain orientation and linker design. SPR revealed affinities of 27.8 nM for EGFRvIII and 15.6 nM for CD3ε, with no cross-reactivity to wild-type EGFR, indicating high target selectivity 69. The fully human format reduces immunogenicity risk and simplifies manufacturing, facilitating clinical translation and scalable production. Functionally, the BsAb promotes immunological-synapse formation between T cells and tumor cells, enabling efficient T-cell redirection and activation. Functional validation showed that hEGFRvIII-CD3 robustly activates both CD4⁺ and CD8⁺ T cells within EGFRvIII-positive microenvironments, augments T-helper-1 cytokine release, and supports repeated rounds of expansion. Under heterogeneous EGFRvIII expression, the BsAb effectively induced T-cell lysis of glioma cell lines and patient-derived samples, with half-maximal effective concentrations for target-cell lysis as low as 6.9-13.3 ng/mL; both CD4⁺ and CD8⁺ T cells exhibited independent cytolytic activity. *In vivo*, intravenous administration enabled BBB transit and intratumoral accumulation in orthotopic models; in patient-derived xenografts, survival was significantly prolonged, with 8/10 mice surviving >100 days, and complete remissions were achieved in subcutaneous models 69, 70. Histology demonstrated extensive tumor necrosis with dense T-cell infiltration, consistent with immune reprogramming.

On the strength of these preclinical data, clinical translation is accelerating. Two EGFRvIII-targeted CD3 BsAbs have entered phase I evaluation. AMG596 (Amgen), administered by continuous intravenous infusion, is being assessed as monotherapy in newly diagnosed and recurrent GBM and in combination with the PD-1 inhibitor AMG404 (NCT03296696) 71. In this trial, 30 patients were enrolled; among the evaluable subset (reported as seven patients), preliminary activity included an objective response rate (ORR) of 12.5% (1/8, 95% CI 0.3%-52.7%) and a disease-control rate of 37.5% (3/8, 95% CI 8.5%-75.5%). Treatment-related adverse events (TRAEs) occurred in all patients; grade ≥3 TRAEs were observed in 50%, including neurologic events such as headache (14%) and depressed consciousness (14%). No CRS or dose-limiting toxicities were reported; pharmacokinetic/pharmacodynamic and CNS-penetration data have not yet been disclosed 72. The BRiTE study (NCT04903795) integrates an anti-EGFRvIII antibody clone (139) and an anti-CD3 antibody (28F11) with peripheral T-cell reinfusion to enhance CD8⁺ T-cell infiltration and improve intratumoral delivery of immune effectors 73, 74. Planned enrollment is 18 patients; the study has not yet initiated and aims to explore a combined BiTE plus activated-T-cell strategy 72. The design leverages the "guiding" effect of activated T cells to facilitate BsAb transit across the BBB, thereby increasing intratumoral pharmacodynamic intensity.

Potential risks warrant attention, particularly immune-effector-cell-associated neurotoxicity syndrome, which may arise from nonspecific T-cell infiltration and inflammatory cytokine release. Prophylaxis or intervention with agents that block lymphocyte trafficking, such as natalizumab, has been proposed as a theoretical mitigation strategy 75. In parallel, a wild-type EGFR-based approach—EGFR-BaT (cetuximab-coated T cells with adjunct OKT3 stimulation)—has entered phase I testing (NCT03344250); in the first-dose cohort, none of four patients experienced dose-limiting toxicity, suggesting an initial safety signal 74, 76.

EGFRvIII×CD3 BsAbs demonstrate compelling translational potential in GBM. By targeting a tumor-specific antigen, they mitigate off-tumor toxicity and, as "off-the-shelf" biologics, circumvent the manufacturing lead times and access constraints inherent to individualized cell therapies. Fully human construction further reduces immunogenicity and enhances generalizability and safety. A current Good Manufacturing Practice -compliant process using a Chinese hamster ovary-cell expression system has been established, and clinical-grade product has been manufactured at Duke University's MPACT facility, providing end-to-end support for phase I advancement. Future studies should prioritize synergy with ICIs, optimization of BBB transit and intratumoral distribution, and deeper investigation of EGFRvIII-negative clonal escape to achieve broader and more durable responses.

### NKG2D-EGFR Bispecifics: Ligand-Independent Targeting

**Figure 7:**
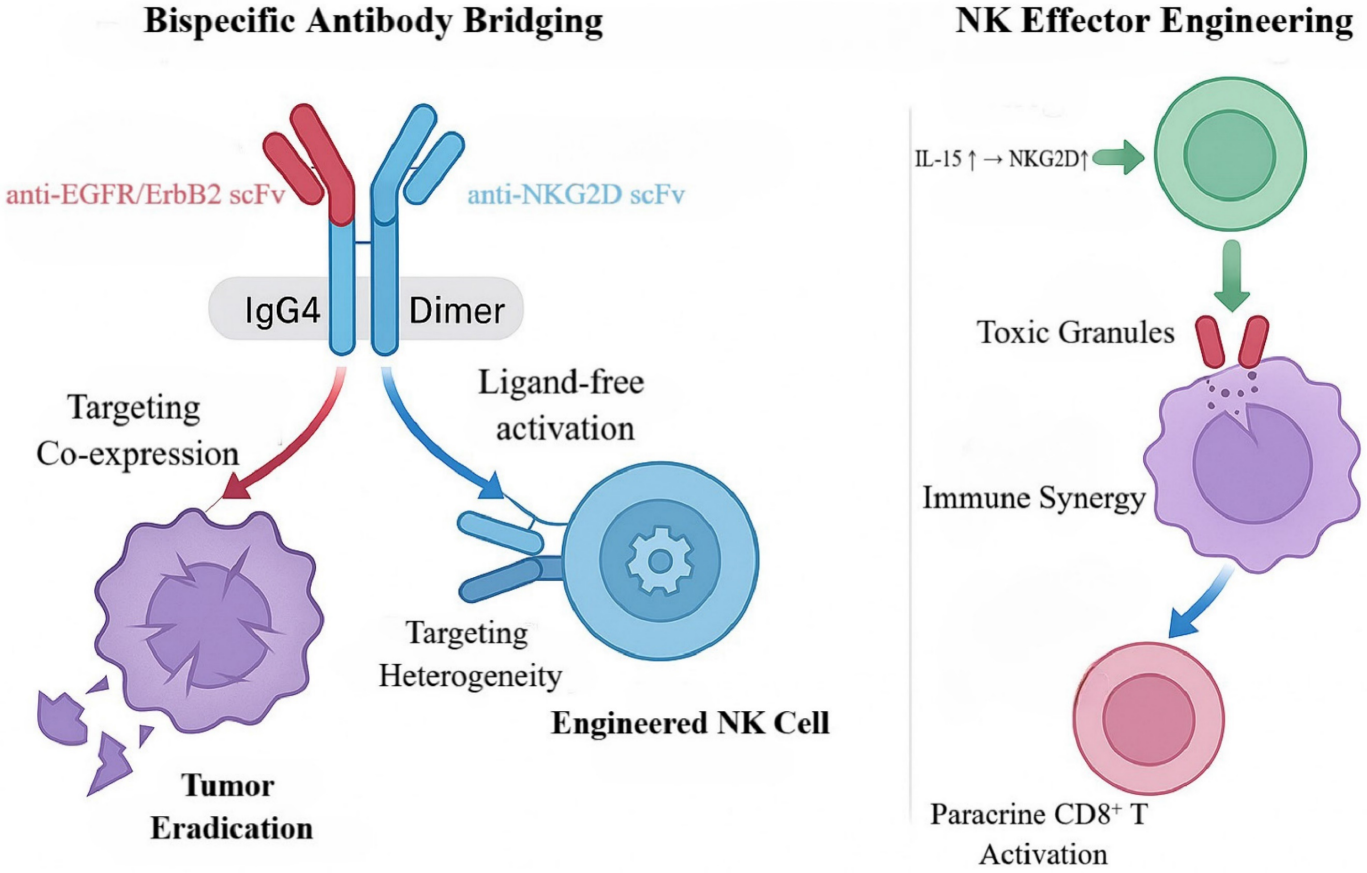
** Mechanism of bispecific antibody-bridged engineered NK cells for synergistic tumor killing.** This schematic illustrates the innovative mechanism by which an EGFR/ErbB2- and NKG2D-targeting bispecific antibody bridges engineered NK cells to synergistically eliminate tumors. The IgG4 dimer-based bispecific antibody simultaneously engages tumor antigens and NK cell receptors, enabling ligand-independent activation. The engineered NK cells enhance their cytotoxic activity through IL-15 autocrine signaling and further activate CD8⁺ T cells via paracrine mechanisms, thereby synergistically overcoming tumor heterogeneity and achieving complete eradication.

In parallel with T-cell-redirecting strategies, BsAbs that harness NK cells as effectors open a complementary avenue for GBM immunotherapy. Natural killer group 2D (NKG2D) is a principal activating receptor broadly expressed on NK cells and cytotoxic lymphocytes (for example, CD8⁺ T cells); it recognizes stress-inducible ligands on tumor cells (e.g., MICA/B and ULBP1-6) and triggers potent cytotoxicity 77. In GBM, however, tumor cells frequently downregulate or shed NKG2D ligands to evade immune surveillance, creating a recognition barrier for approaches relying on native NKG2D-ligand interactions 78. Concurrently, EGFR and its family member ERBB2 (HER2) are often co-expressed or amplified in GBM, driving proliferation, invasion, and therapeutic resistance 79. Together, impaired immune surveillance and sustained oncogenic signaling constitute dual obstacles within the TME. This rationale supports artificial reconstruction of the immune synapse by forcibly bridging NKG2D-CAR-engineered NK cells to EGFR/ERBB2-positive tumor cells, thereby overcoming immune escape and addressing intratumoral heterogeneity (Figure 7).

To counter this evasion, Anne K. et al. designed a series of bispecific killer-cell-bridging antibodies (NKAB-EGFR and NKAB-ERBB2) that link NKG2D-CAR NK cells to EGFR or ERBB2, enabling ligand-independent, precision targeting 80. These BsAbs employ an IgG4-Fc scaffold that tethers anti-EGFR/ERBB2 and anti-NKG2D single-chain antibodies into a stable homodimer, enhancing molecular stability and cell-cell crosslinking. Mechanistically, the antibodies simultaneously engage EGFR/ERBB2 on tumor cells and the NKG2D-CAR on NK cells, establishing close-range immune synapses that directly activate CAR-NK cells irrespective of endogenous NKG2D-ligand expression, thereby overcoming target-recognition loss 81. To further potentiate NK-cell function, the investigators generated engineered NK-92 cells via lentiviral transduction to co-express an NKG2D-CAR and an interleukin-15 (IL-15) superagonist (RD-IL15); the former confers target recognition, whereas the latter sustains cell fitness via autocrine signaling, upregulates NKG2D, and induces paracrine activation of CD8⁺ T cells—together building a multidimensional immune-synergy platform 80.

In mixed tumor-cell models that recapitulate GBM antigen heterogeneity, single-agent NKAB-EGFR or NKAB-ERBB2 lysed only their respective antigen-positive subclones, with lysis rates of ~20%-30% 80. In combination, the BsAbs covered a broader clonal repertoire, increasing lysis to >50% and mitigating the limitations imposed by antigen heterogeneity 82, 83. Additional experiments showed that engineered NK cells bridged by BsAbs released higher levels of cytotoxic mediators and elicited vigorous CD8⁺ T-cell activation and proliferation via RD-IL15-mediated paracrine signaling and cooperation among multiple effector subsets. Relative to single-antibody groups, the combination more than doubled tumor-cell clearance (p<0.01) and eliminated nearly 80% of tumor burden in heterogeneous models, offering a practicable strategy to counter GBM antigen escape.

*In vivo*, BsAbs combined with NKG2D-CAR NK cells significantly enhanced tumor clearance and prolonged survival, demonstrating durable efficacy and the feasibility of an "off-the-shelf" cellular-immunotherapy approach 81, 84. Challenges remain: low-level EGFR expression in normal tissues raises on-target/off-tumor toxicity concerns; brain accumulation after intravenous dosing is constrained by the BBB; and sustained IL-15 signaling may promote NK-cell exhaustion. These issues underscore the need to optimize delivery systems and regulate transgene expression to improve safety and durability. Overall, BsAbs targeting NKG2D together with EGFR/ERBB2 re-engineer immune recognition within the TME by bridging engineered NK cells to oncogenic drivers and—when coupled with IL-15-based activation—achieve synergistic effects with broad antigen adaptability, thereby expanding therapeutic options for GBM immunotherapy 85. Future studies should prioritize systematic assessment of off-tumor toxicity and elucidation of immune-tolerance mechanisms to support clinical translation.

### Universal NKG2D Recognition: Overcoming Heterogeneity

Beyond selectively engaging NK cells, an innovative therapeutic paradigm couples the broad ligand recognition of NKG2D with T-cell redirection. Baugh and colleagues developed an NKG2D×CD3 BiTE whose core innovation is architectural: the extracellular domain of NKG2D substitutes for the conventional scFv as the tumor-binding arm 86. This design enables simultaneous recognition of all eight human NKG2D ligands—MICA, MICB, and ULBP1-6—thereby addressing the pronounced heterogeneity of ligand expression in GBM.

*In vitro*, exposure to 1 nM BiTE for 72 h significantly activated both CD4⁺ and CD8⁺ T cells (increasing the CD69⁺ fraction by >40%) and triggered robust release of GZMB and perforin together with a >3-fold rise in IFN-γ. Correspondingly, U87 cell viability declined to ~40% versus >85% in controls. Notably, temozolomide (TMZ) and low-dose radiotherapy, via induction of the DNA-damage response, further upregulated NKG2D-ligand expression, enabling the BiTE at 0.1 nM to achieve an ≈50% increase in cytotoxicity. These findings support chemoradiotherapy as a priming, sensitizing step preceding BiTE administration and motivate a sequential immuno-combination strategy 81.

The approach also shows activity against glioma stem cells (GSCs), a principal reservoir of therapeutic resistance. The NKG2D×CD3 BiTE gene was inserted into OVG207 to generate an “armed” OVs, G207-NKG2D BiTE 87. Upon infecting permissive tumor cells, the virus drives local, sustained secretion of functional BiTE molecules. Although GSCs display marked resistance to G207 infection (infection rate <5%), their stable, high-level NKG2D-ligand expression confers intrinsic susceptibility to the BiTE. In co-culture, soluble BiTE induced ~60% GSC death, and BiTE released from neighboring G207-NKG2D BiTE-infected cells produced a similar bystander effect, reducing GSC viability to ~45% 88-90.

This fusion strategy delivers three synergistic mechanisms: (1) direct oncolysis of infected cells by the OVs; (2) BiTE-mediated T-cell elimination of treatment-resistant GSCs; and (3) chemoradiotherapy-induced upregulation of NKG2D ligands to heighten BiTE sensitivity 91. In animal models, the combination significantly curtailed tumor progression and increased intratumoral immune infiltration, with CD8⁺ T cells rising by ~1.8-fold—consistent with durable antitumor immunity and microenvironmental remodeling 88. Collectively, this integrative platform offers a path to overcome GSC-driven resistance while providing a structural solution for efficient BiTE delivery and amplification within GBM's complex ecosystem.

### EPHA2/A3 Co-inhibition: Suppressing GSC Stemness

Beyond modulating immune effector cells or growth factor receptors, directing bispecific strategies toward receptors on GSCs offers a means to address GBM recurrence at its source. Ephrin type-A receptor 2 (EPHA2) and Ephrin type-A receptor 3 (EPHA3)—key Eph receptor tyrosine kinases—govern cell migration, adhesion, and maintenance of stemness and are implicated in tumor stemness and therapeutic resistance across malignancies 92, 93. In GBM, particularly in recurrent glioblastoma (rGBM), EPHA2 and EPHA3 show conspicuous co-expression, constituting a signaling axis that fuels tumor evolution and therapeutic escape 94. This co-expression is enriched in GSC-like subpopulations and colocalizes with canonical stemness markers such as SOX2 and BMI1, implicating this axis in maintaining stemness while promoting recurrence 95.

On this molecular basis, investigators have proposed dual-target inhibition to counter the stem cell-driven biology of rGBM. High-dimensional cytometry by time of flight revealed enrichment of EPHA2/EPHA3 double-positive cells in patient-derived GSCs from recurrent tumors, correlating with increased clonogenic capacity and worse clinical outcomes 96. Analyses of The Cancer Genome Atlas further support these observations: patients with high EPHA2/EPHA3 expression exhibit markedly shorter median survival, underscoring the therapeutic relevance of this axis. To test targetability, Sheila K. S. and colleagues developed an EPHA2/EPHA3 bispecific antibody (EPHA2/A3 BsAb) designed to impose coordinated interference on both receptors—inducing functional inhibition and downregulation. In cell-based assays, antibody engagement of EPHA2 triggered receptor phosphorylation followed by internalization and degradation, leading to a pronounced reduction in EPHA2 protein abundance. By contrast, although EPHA3 phosphorylation was not induced, surface EPHA3 was significantly suppressed, consistent with steric occupancy and conformational interference that together disrupt downstream signaling 97.

At the signaling level, EPHA2/A3 BsAb treatment rapidly inhibited pro-tumorigenic nodes, including serine/threonine kinase (AKT) and extracellular signal-regulated kinases 1/2. Notably, prolonged exposure did not elicit compensatory upregulation of other Eph family members, indicating favorable target specificity. Functionally, the BsAb reduced clonogenicity and stem-cell frequency in rGBM cells and induced a differentiated phenotype, as evidenced by increased Glial fibrillary acidic protein and Microtubule-associated protein 2 expression—supporting a dual mechanism that attenuates maintenance of stemness while activating differentiation programs 98. Proof-of-concept *in vivo* studies further substantiated therapeutic potential. Despite suboptimal intracranial exposure attributable to molecular size constraints, the BsAb nevertheless downregulated receptor expression and stemness markers within orthotopic tumors and significantly reduced tumor burden 19. These data indicate that local, coordinated suppression of EPHA2/EPHA3 signaling together with induction of tumor-cell differentiation can selectively deplete the core stem-like compartment in rGBM.

In summary, the EPHA2/A3 BsAb selectively impairs survival and expansion of rGBM populations enriched for GSC features by simultaneously perturbing the expression and function of two pivotal stemness receptors. On one hand, it induces EPHA2 phosphorylation and rapid internalization-degradation; on the other, it suppresses EPHA3 surface expression via steric interference—yielding complementary receptor downregulation. Collectively, downstream signaling is durably suppressed, GSC stemness is eroded, and tumor cells are driven toward differentiation 97. By concurrently diminishing stemness maintenance and enhancing differentiation propensity, this bispecific approach addresses limitations of single-target interventions. Demonstrated target engagement and efficacy in cellular and animal models support its feasibility and translational potential as a stemness-focused therapy for rGBM.

### IL-13Rα2 BiTE: Establishing TRM Against Antigen Loss

Shifting from tumor-intrinsic receptor targeting to mobilizing adaptive immunity, BsAbs that engage IL-13Rα2 on tumor cells and CD3 on T cells open a distinct therapeutic avenue. IL-13Rα2, a high-affinity decoy receptor, is minimally expressed in normal brain but overexpressed in more than 50% of GBM; abundance correlates with higher grade, invasiveness, and poor prognosis 99. Beyond competitively modulating the IL-13/IL-4 axis, IL-13Rα2 has emerged as one of the most tractable TAAs in GBM. In parallel, the CD3ε chain on T cells serves as a pivotal hub for activation signaling and an attractive trigger for precise immune engagement 74. Accordingly, an IL-13Rα2×CD3 BiTE can couple tumor recognition with effector activation in a “target-effector” dual-binding mode, offering a strategy to overcome immune escape driven by loss of MHC class I (MHC-I).

Guided by this immuno-engineering concept, Katarzyna C. P. et al. constructed a BiTE that simultaneously and specifically recognizes IL-13Rα2 and CD3ε. Via linked scFvs, the molecule bridges tumor cells and T cells to induce MHC-independent immunological-synapse formation, thereby enhancing T-cell activation while circumventing constraints of classical antigen presentation. In orthotopic, immunocompetent transplant models and in genetically engineered mouse models, the BiTE exhibited a two-pronged activation mechanism: selective engagement of IL-13Rα2⁺ tumor cells and CD3-dependent release of IFN-γ and tumor necrosis factor-α (TNF-α), together with GZMB-mediated cytotoxicity. Even within the profoundly immunosuppressive GBM TME, the BiTE achieved >60% in-vitro tumor-cell lysis, and its activity was retained despite T-cell exhaustion markers (PD-1, TIM-3, Lag-3) or concomitant glucocorticoids, indicating functional stability and resistance to exhaustion 100, 101.

*In vivo*, the BiTE not only directly cleared tumor cells but also remodeled the TME. Flow cytometry and single-cell RNA sequencing demonstrated a >4-fold increase in intratumoral CD8⁺ T-cell infiltration, expansion of tissue-resident memory T cells, and a significant reduction in immunosuppressive subsets such as monocytic myeloid-derived suppressor cells (Mo-MDSCs). Critically, the immune effect displayed durability and memory: survivor mice remained fully protected upon rechallenge with IL-13Rα2-negative tumors, accompanied by sustained intracerebral enrichment of CD8⁺ T cells and robust cytokine up-regulation—features consistent with vaccine-like immunological memory 100.

To surmount the physical delivery barrier posed by the BBB, the investigators deployed engineered neural stem cells (NSCs) as carriers that secreted functional BiTEs within the brain for >7 days 102. This approach significantly prolonged survival across multiple GBM models (for example, extending median survival from 17 to 33 days in the SMA560-IL13RA2 model) without overt neurotoxicity. Immunohistochemistry confirmed increased intratumoral GZMB and CD69⁺ T-cell densities, supporting effective activation of T-cell effector function 100. Nonetheless, ~60% of animals exhibited “tumor-bearing survival,” suggesting persistence of immunosuppressive myeloid populations and underscoring the complexity of the GBM microenvironment as a barrier to complete remission 103, 104.

Although IL-13Rα2 is stably expressed in a majority of GBMs, spatial heterogeneity may permit immune escape 105, 106. Interspecies differences (for example, ~47% homology between murine and human CD3-binding domains) and the intrinsically short half-life of BiTEs further limit translational efficiency 107. Strategies to improve pharmacokinetics and tissue retention include humanization, Fc fusion or albumin binding to extend systemic exposure, and intrathecal administration or nano-delivery systems to optimize CNS distribution 107. Collectively, an IL-13Rα2×CD3 BiTE constitutes a multifunctional platform that integrates high specificity, potent immune activation, and favorable translational attributes, providing a strong preclinical rationale for precision therapy in GBM.

### IL-13Rα2/CD16 NK Engager: Reversing Immunosuppression

Given the central role of innate immune cells in tumor surveillance, bispecific strategies that activate NK cells constitute a complementary avenue for GBM immunotherapy. An IL-13Rα2×CD16 bispecific killer engager (BiKE)—often formulated as a TriKE when incorporating cytokine payloads—exemplifies this approach. CD16 (FcγRIII) is a low-affinity Fc receptor expressed on NK cells, macrophages and neutrophils, and serves as a key activating receptor for ADCC, inducing effector-cell degranulation and secretion of pro-inflammatory cytokines 108. In GBM, IL-13Rα2 is highly and selectively expressed, providing a precise anchor for targeted therapy. Together, IL-13Rα2 and CD16 establish complementary recognition-activation circuitry within the TME: IL-13Rα2 confers tumor-specific binding, whereas CD16 triggers NK-cell cytotoxicity. Accordingly, a BsAb co-targeting IL-13Rα2 and CD16 is designed to redirect NK-mediated ADCC for selective elimination of IL-13Rα2-positive GBM cells and to enhance resilience against an immunosuppressive milieu.

This engager adopts a single-chain architecture characteristic of TriKEs, comprising three functional modules: a single-domain antibody specific for CD16, an IL-15 linker segment to augment NK-cell survival and proliferation, and an scFv that binds IL-13Rα2 109-111. Through spatial reprogramming, it elicits two synergistic effects: (1) the CD16-binding domain activates NK-cell ADCC, promoting release of PRF1 and GZMB and secretion of cytokines such as IFN-γ and tumor necrosis factor-α (TNF-α) 112; and (2) the IL-15 moiety enhances NK-cell persistence, expansion and effector function, thereby helping to counter GBM-associated immunosuppression 113. *In vitro*, the engager mediates high-efficiency lysis of IL-13Rα2-positive GBM cells, with cytolysis rates exceeding 80%.

Preclinical studies further show that the engager increases NK-cell cytotoxicity against GBM cells with high IL-13Rα2 expression, induces mitochondrial-pathway apoptosis, and markedly elevates CD25/CD69 expression and caspase-3 activity. Although activity is detectable against cells with lower IL-13Rα2 expression, efficacy diminishes as antigen density decreases, underscoring the importance of target abundance. In animal models, co-administration of the engager with human NK cells significantly prolongs survival, accompanied by increased intratumoral NK-cell infiltration and higher numbers of caspase-3-positive tumor cells, consistent with partial reversal of TME immunosuppression. A practical advantage of this strategy is its reliance on NK cells rather than T cells, obviating antigen presensitization or HLA matching and conferring a very low risk of graft-versus-host disease. Remaining hurdles include antigen heterogeneity, interference by immunosuppressive factors and limited BBB permeability; moreover, IL-15-driven activation may provoke systemic immune stimulation 114. Despite these challenges, IL-13Rα2×CD16 bispecific molecules continue to show meaningful therapeutic potential in recurrent GBM.

In conclusion, novel immunotherapies represented by BsAbs are profoundly transforming the treatment landscape of GBM (Table 1). Although challenges remain ahead, these research achievements undoubtedly bring unprecedented hope and opportunities for us to ultimately conquer this "king of cancers."

## Exploration of New Technologies

To address these constraints comprehensively, investigators are advancing a suite of delivery technologies and antibody-engineering strategies (Table 2). These efforts seek to surmount limited transport across the BBB, intratumoral heterogeneity, treatment-limiting toxicities, and inadequate pharmacodynamic durability.

Current directions coalesce around four themes: (1) breaching the BBB—for example, transferrin-receptor (TfR) shuttle antibodies, MMP-2-responsive “smart” nanoplatforms, NSCs carriers, and biomimetic nanocarriers with homotypic (“tumor-self”) recognition; (2) countering heterogeneity—by designing formats that co-engage multiple TAAs and/or effector-cell receptors; (3) improving safety—through controlled-release systems, Fc-domain-silencing mutations, and fine-tuning of affinity and interdomain geometry to restrict on-target/off-tumor activation; and (4) enhancing potency and durability—by fusing cytokines or costimulatory modules to sustain antitumor activity. The subsequent sections critically appraise these strategies and their developmental trajectories for overcoming translational bottlenecks of BsAbs.

### TfR-BsAb Fusion: Enhanced BBB Penetration Avoiding Competition

**Figure 8:**
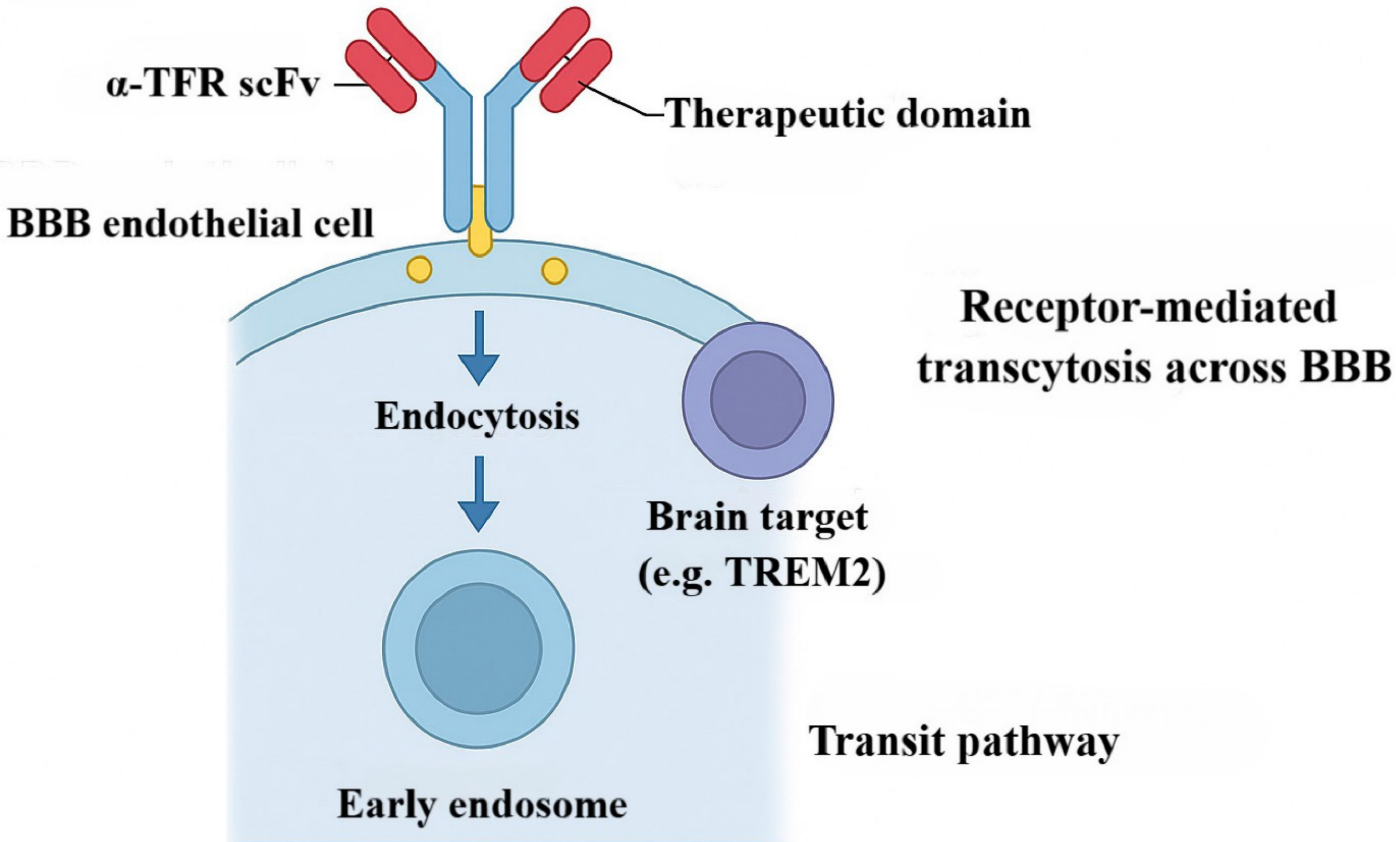
** Mechanism of TfR-mediated transcytosis of BsAbs across the blood-brain barrier.** This illustration reveals the delivery pathway by which a bispecific antibody traverses the blood-brain barrier via transferrin receptor (TfR)-mediated transcytosis. Utilizing its α-TfR scFv domain, the antibody specifically binds to cerebrovascular endothelial cells, is internalized into early endosomes, and undergoes intracellular trafficking to complete transcellular transport. Ultimately, it releases its therapeutic domain into the brain parenchyma to precisely target the lesion, offering a targeted therapeutic strategy for CNS disorders.

Among strategies to traverse the BBB, exploiting the TfR is particularly promising. TfR-mediated delivery has emerged as a leading approach to overcome BBB constraints and improve CNS-targeted therapy 116, 117. The core concept is to engineer BsAbs with one arm against the disease-relevant antigen and the other recognizing TfR on brain microvascular endothelium. For example, in Alzheimer's disease, investigators fused a therapeutic anti-TREM2 antibody with a TfR-binding domain (for example, scFv8D3). The resulting BsAb harnesses receptor-mediated transcytosis (RMT) to cross the BBB: after TfR engagement and entry into early endosomes, the antibody is ferried across endothelial cells and released into the parenchyma 118. Format optimization—such as an IgG-(scFv)₂ architecture—substantially enhances TfR engagement and BBB transit, increasing intracerebral antibody exposure by orders of magnitude and enabling *in vivo* molecular imaging of neuroinflammatory targets 117, including TREM2 PET readouts (Figure 8) 119, which has advanced diagnostic strategies for neurodegenerative disease 120, 121. Notably, recent mechanistic and engineering advances in TfR targeting lay the groundwork for translation to the even more challenging setting of brain tumors.

Mechanistically, TfR-enabled transport relies on RMT: engineered antibody fragments bind TfR, trigger endocytosis, traverse the endothelial layer within vesicles, and are released into brain tissue 122, 123. Recent work indicates that monovalent designs (for example, VEGF-Trap/moAb4) can avoid excessive TfR internalization and lysosomal routing, thereby prolonging residence time in brain tumors 23. Key advances include affinity tuning (equilibrium dissociation constants, K_D, in the 10-100 nM range) to balance transport efficiency with off-target risk; creation of dual-targeting systems (for example, heterodimers engaging TfR/TfR2); and engineering of pH-sensitive antibodies that exploit the acidic tumor milieu for conditional activation, improving specificity while reducing hepatotoxicity 23. The platform has also been extended to deliver diverse modalities—such as siRNA, CRISPR/Cas9 cargos, and immunocytokines—underscoring its broad applicability.

Despite progress in neurodegeneration, applications in brain tumors—particularly GBM—remain relatively limited. Anti-angiogenic regimens widely used in GBM can remodel BBB architecture and further restrict drug entry 124. To address this barrier, researchers developed a monovalent TfR-BsAb (VEGF-Trap/moAb4) that, for the first time, achieved efficient active delivery under conditions of BBB remodeling 122. This advance rests on three pillars. First, target engagement: the Ab4 antibody, identified by phage display, binds a distal apical TfR epitope, minimizing competition with endogenous transferrin and preserving iron homeostasis while maintaining delivery efficiency 125. Second, construct engineering: an aflibercept-mimetic VEGF-capturing domain is fused to Ab4, and Fc-region mutations L234A/L235A/P329G (LALAPG) are incorporated to silence Fcγ-receptor-mediated activity and reduce risks of antibody-dependent cellular phagocytosis and CDC 126. Third, transport routing: monovalent binding triggers clathrin-mediated endocytosis while avoiding ESCRT complex activation; cargo thereby bypasses lysosomes and enters Rab4/Rab11-regulated rapid-recycling pathways 127. This design maintains TfR surface homeostasis and markedly increases transport throughput. Compared with conventional bivalent BsAbs, the monovalent construct reprograms TfR-mediated endocytosis and recycling to achieve both efficient BBB transit and anti-angiogenic activity, demonstrating superior potential for brain-tumor therapy. Ongoing work is exploring advanced solutions and combinations to further enhance efficacy and translational readiness.

Combination regimens are also emerging. In preclinical studies, TfR-targeted nanoparticles loaded with TMZ and combined with PD-1 blockade increased tumor-growth inhibition to 82% while significantly reducing toxicity 128, 129. Additional approaches are probing synergy between TfR-mediated delivery and OVs or CAR-T therapies to augment intratumoral accumulation and *in situ* immune activation. Future directions include individualized delivery systems (parameter optimization using patient-derived organoids) and third-generation TfR antibodies featuring cleavable linkers and Fc-silencing mutations; together, these advances position TfR-targeted delivery as a brain-directed platform with strong translational prospects. From a regulatory standpoint, the US Food and Drug Administration (FDA) has issued guidance on brain-targeted delivery systems, which is expected to accelerate clinical translation.

### MMP-2-Triggered Nanocapsule: Spatiotemporal BsAb/Ferroptosis Synergy

**Figure 9:**
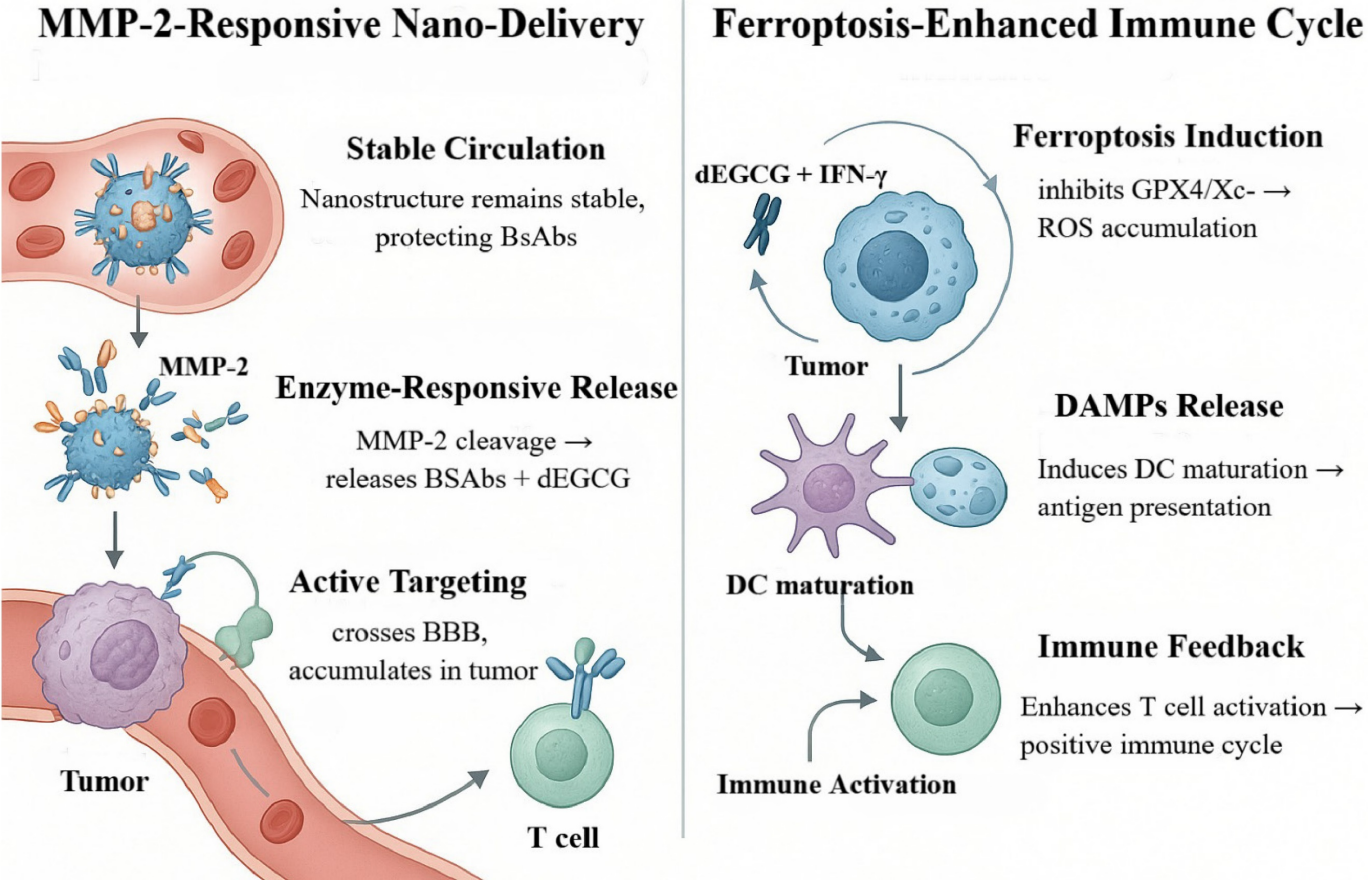
** Mechanism of MMP-2-responsive nanosystem for synergistic ferroptosis-immunity antitumor therapy.** This schematic illustrates how an MMP-2-activated nanosystem enhances brain tumor treatment. The hyaluronic acid-targeted nanocarrier crosses the blood-brain barrier and accumulates in the tumor, where it responds to MMP-2 to release a bispecific antibody (BsAb) and an epigallocatechin gallate derivative (dEGCG). Together with T cell-derived IFN-γ, dEGCG induces ferroptosis by inhibiting the GPX4/Xc⁻ pathway, leading to damage-associated molecular pattern (DAMP) release. This promotes dendritic cell (DC) maturation and antigen presentation, establishing a positive feedback loop that enhances T cell activation and synergistically inhibits tumor growth.

Orthogonal to RMT, an alternative strategy leverages biochemical cues unique to the TME to achieve conditionally triggered, spatially precise drug release. Beyond TfR-directed mechanisms, enzyme-responsive “smart” delivery systems provide a complementary paradigm for directing BsAbs to GBM. A recently developed MMP-2-responsive nanoplatform (S-biAb/dEGCG@NPs) substantially increases intratumoral accumulation and therapeutic efficacy of antibodies through multimodal synergy, offering a forward-looking solution to delivery barriers in GBM therapy 130. This modular platform centers on the MMP-2-cleavable peptide PLG*LAG as the response unit; relative to conventional sequences, its proteolytic efficiency is increased 3.2-fold and it triggers efficient release when MMP-2 exceeds 50 nM, achieving ~60% cumulative release within 12 h—substantially outperforming nonresponsive carriers 131, 132. Capitalizing on elevated MMP-2 in GBM, the nanostructure incorporates the substrate peptide PLGLAG and covalently couples hyaluronic acid to dEGCG (Figure 9) 65. The construct exhibits high circulatory stability, protecting encapsulated BsAbs from degradation and limiting off-target exposure. Upon tumor accumulation, MMP-2-mediated scission rapidly disassembles the nanoparticles, enabling spatiotemporally controlled release of BsAbs and dEGCG and thereby enhancing tumor selectivity and local bioactivity 133. In preclinical studies, this strategy achieved intratumoral drug concentrations up to ~38-fold higher than in peripheral tissues, extended survival 2.8-fold, and reduced tumor volume by 76% in U87-Luc xenografts 65.

Targeting specificity is further reinforced by HA, which functions not only as the structural scaffold but also as an active ligand for CD44 overexpressed on GBM cells. HA-CD44 interactions facilitate BBB transit and selective accumulation with deep parenchymal penetration, establishing the spatial basis for subsequent enzyme-triggered release 63. Contemporary designs introduce hierarchical responsiveness—for example, pH-sensitive layers to promote endosomal escape and reactive-oxygen-species-responsive motifs to sharpen delivery within oxidative niches—thereby improving targeting fidelity and release control 134, 135.

Functional synergy is a defining feature of this platform. Spatiotemporally released BsAbs can bridge B7-H3 on tumor cells with CD3ε on T cells, activating effector functions and driving tumor-specific cytotoxicity. In parallel, dEGCG cooperates with tumor-infiltrating lymphocyte (TIL)-derived IFN-γ to potentiate ferroptosis by suppressing GPX4 and inhibiting system x_c⁻, leading to sustained lipid-peroxide accumulation and regulated cell death 65. This process upregulates acyl-CoA synthetase long-chain family member 4, increases lipid peroxidation by up to 4.3-fold, synergizes with T-cell cytotoxicity, downregulates SLC3A2/SLC7A11, and promotes M1 macrophage polarization—collectively remodeling the immune milieu 64, 136. The consequent release of damage-associated molecular patterns (DAMPs) promotes dendritic-cell maturation and antigen presentation, further amplifying T-cell activation to establish a positive-feedback loop of “immune activation → ferroptosis intensification → antigen release → T-cell re-activation,” thereby enhancing both durability and magnitude of antitumor immunity 64, 137.

This system demonstrates robust intratumoral accumulation and therapeutic activity across multiple GBM models, yielding significant tumor-growth inhibition and survival extension. The platform has advanced to the IND-enabling stage and, in combination with anti-PD-1 antibodies or focused ultrasound, further increases T-cell infiltration and BBB penetration 138, 139. Looking ahead, priorities include personalized dosing paradigms, theranostic integration, and development of multi-trigger systems. The platform has reportedly received FDA orphan-drug designation for recurrent GBM, paving the way for clinical translation. By integrating targeted delivery, enzyme-responsive release, and immunologic co-activation—while avoiding many limitations of systemic administration—this strategy offers a promising route and translational opportunity for precision immunotherapy in GBM.

### NSC BiTE Factories: Sustained Activity Against Exhaustion

Compared with synthetic nanocarriers, leveraging living cells endowed with innate tumor tropism as delivery vehicles offers a compelling route to sustain high intratumoral concentrations of therapeutic antibodies. Beyond nanoplatforms and BBB-shuttle antibody-engineering strategies, cell-based delivery is emerging as an important means to enhance BiTE therapy. In this context, investigators have engineered NSCs as vehicles to overcome delivery bottlenecks for BiTEs in GBM. The approach exploits two biological attributes of NSCs: first, chemotaxis toward stromal cell-derived factor 1 (SDF-1/CXCL12), enabling efficient homing to tumor regions (migration efficiency >85%); and second, functioning as living secretory factories that continuously produce therapeutic molecules—such as an IL-13Rα2×CD3 BiTE—at ~5-10 ng per 10^6 cells per day while maintaining metabolic activity under profound hypoxia 140, 141. Taking advantage of NSC tropism for brain tumors, modified NSCs stably secrete IL-13Rα2×CD3 BiTEs and, after intracranial injection, actively migrate to and accumulate within tumor territories. Dynamic MRI shows that within 24-48 h post-injection, NSCs form dense foci in the tumor hemisphere and secrete functional BiTEs locally for >7 days, markedly prolonging on-site exposure and overcoming the rapid systemic clearance observed with peripheral dosing 101, 142, 143.

This platform integrates cellular homing with *in situ* sustained release, yielding multiple antitumor effects in GBM. It achieves intratumoral BiTE concentrations that exceed circulating levels by orders of magnitude, thereby effectively activating cytotoxic T cells; simultaneously, co-secretion of immunomodulators (for example, TGF-β inhibitors, IL-12) reverses immunosuppression, reducing M2-polarized macrophages from ~45% to ~12%; in parallel, secretion of thrombospondin-1 (TSP-1) suppresses aberrant angiogenesis and improves intratumoral perfusion, creating favorable conditions for drug delivery 101. *In vitro*, NSC-secreted BiTEs robustly activate patient-derived CD3⁺ T cells—including peripheral-blood T cells and TIL—and retain function despite high expression of exhaustion markers (PD-1, TIM-3, LAG-3). By bridging IL-13Rα2⁺ tumor cells and CD3ε on T cells, the BiTE induces T-cell activation (CD69/CD25 upregulation), GZMB release, and production of inflammatory cytokines (IFN-γ, TNF-α), culminating in tumor-specific lysis. This effect is strongly IL-13Rα2-dependent yet relatively insensitive to T-cell exhaustion phenotypes, indicating preserved activity within an immunosuppressive TME 144.

Ongoing enhancements prioritize prolonging cell persistence, improving safety, and optimizing combinations. For instance, hypoxia-inducible factor-1α upregulation increases hypoxia tolerance, extending NSC survival in tumor cores from ~1 week to ~3 weeks 145; inclusion of an inducible suicide switch enables >95% ablation of transplanted cells within 4 h 146; and combinations with CAR-T cells or immune-checkpoint inhibitors further extend survival and mitigate T-cell dysfunction. A newly developed “dormancy-activation” control pharmacologically modulates the mammalian target of rapamycin pathway to tune the NSC cell cycle, increasing therapeutic efficacy by ~3.2-fold 147. In preclinical xenograft models (e.g., GBM6, GBM12), a single intratumoral dose of engineered NSCs combined with T-cell infusion—delivered intracranially or intravenously—significantly prolonged survival, substantiating therapeutic potential 101. Notably, the NSC platform is off-the-shelf, obviating individualized manufacturing and enhancing clinical feasibility.

From a translational standpoint, two NSC-based BiTE therapies have entered phase I clinical testing; preliminary data indicate substantially higher BiTE levels in cerebrospinal fluid than in serum and no apparent cell-related toxicities to date. The FDA has issued guidance for cellular-therapy products, providing a framework for quality control. Future work should prioritize stimulus-responsive NSCs, individualized dosing, and rational combination strategies, while addressing intratumoral heterogeneity, long-term safety, and manufacturing control. Although durability and performance under highly heterogeneous antigen landscapes require further evaluation, NSC-mediated *in situ* BiTE expression establishes a robust foundation for a highly targeted, effective, and low-toxicity immunotherapeutic pathway in GBM.

### GBM-PDTCM@AuNRs: Multimodal Imaging-Guided Therapy

Further emulating biological systems, biomimetic nanotechnology provides an innovative strategy to overcome the BBB and achieve tumor-selective targeting by conferring “self” recognition on drug carriers. Recently, a homology-based (homotypic) nanoplatform was introduced to tackle the dual obstacles of BBB penetration and targeted delivery in GBM. The approach preserves adhesion molecules intrinsic to tumor-cell membranes, such as ICAM-1 and EpCAM, thereby enabling selective recognition and binding to homologous tumor cells 148. Gradient centrifugation and immunoaffinity purification preserve over 85% of critical targeting proteins while minimizing immunogenicity. Using patient-derived GBM cell membranes (GBM-PDTCM) to cloak functionalized gold nanorods (AuNRs), investigators generated a theranostic nanocomplex (GBM-PDTCM@AuNRs) 149. This system leverages membrane-based homotypic recognition, in which retained tumor antigens interact with BBB endothelium, initiating RMT and facilitating efficient barrier crossing 150. Architecturally, the carriers adopt multilayer configurations—comprising a drug-loading core, a pH-responsive polymer intermediate, an outer tumor-cell membrane shell, and an additional penetration-peptide coating—that sequentially navigate biological barriers and culminate in precise intracellular release. *In vitro*, transcytosis efficiency reached 46.97% versus 1.74% for non-brain-derived membranes (e.g., HeLa). Critically, patient-derived membranes preserve native tumor antigenicity and biointerfaces, enhancing homotypic recognition and tumor-selective accumulation within GBM-like microenvironments.

Beyond delivery, the platform integrates multimodal imaging. Co-loading Raman reporters (e.g., p-ATP) and lipophilic near-infrared (NIR) dyes (e.g., DiR, DiI) yields synergistically amplified optical signals within GBM lesions. Functionally, the nanocomplex affords three advantages: (1) tumor-specific targeting significantly reduces off-target toxicity; (2) immune camouflage prolongs systemic circulation to approximately 16 h; and (3) the acidic TME accelerates drug release at rates 5.8-fold higher than in normal tissues. NIR fluorescence delineates tumor cores, whereas highly sensitive surface-enhanced Raman scattering (SERS) identifies infiltrative margins 151, 152. In animal models, the system enabled intraoperative guidance, achieving >95% resection with combined NIR imaging and handheld Raman probes and potentially improving completeness of resection and postoperative outcomes in advanced GBM 150.

Refinements now focus on extending membrane stability beyond 24 h in circulation, establishing microfluidic manufacturing for standardized scale-up, and building patient-specific membrane banks to support individualized therapy. Hybrid-membrane technologies, fusing erythrocyte and tumor membranes, further prolong systemic circulation while preserving targeting specificity 153. The platform has also been adapted for immunotherapy and gene-editing applications, such as CRISPR-Cas9 delivery for *in situ* genome modulation, and for combination regimens with radiotherapy or immune-checkpoint blockade to augment antitumor immunity 154, 155. Therapeutically, AuNRs provide robust photothermal conversion: upon NIR irradiation, biomimetic nanocarriers selectively accumulate in tumor tissue, generate localized hyperthermia, and induce apoptosis or necrosis, thereby suppressing tumor growth and extending survival in orthotopic GBM models. Notably, this photothermal approach achieves tumor-specific efficacy with a favorable safety profile independent of chemotherapeutics, complementing existing regimens.

From a translational perspective, universal and patient-tailored platforms have advanced to IND-enabling studies, with the FDA issuing quality-control guidance. Future directions include developing stimuli-responsive carriers, integrating theranostic systems, and leveraging artificial intelligence to optimize membrane-protein composition—addressing tumor heterogeneity and reducing production costs. Overall, the GBM-PDTCM@AuNR platform, driven by homotypic recognition, integrates BBB penetration, tumor-selective accumulation, diagnostic imaging, intraoperative navigation, and photothermal therapy. By providing a programmable, stage-adaptable delivery system, it offers a generalizable vehicle for diverse payloads—including BsAbs, siRNA, and other macromolecules—with substantial translational promise.

### Armored OV Expressing BiTE: Breaking Immunosuppressive Barriers

Beyond delivery innovations, combining BsAbs with other modalities—most notably OVs—represents a frontier for amplifying antitumor immunity. OV therapy has gained traction in solid tumours. The first licensed agent, talimogene laherparepvec (T-VEC), administered intratumorally, replicates within tumours to lyse infected cells while expressing granulocyte-macrophage colony-stimulating factor (GM-CSF) to prime systemic immunity; in advanced melanoma it improved response rates and durability of benefit 156. This success—enabled by feasible local administration and reprogramming of an immunosuppressive TME—offers a precedent for hard-to-treat entities such as GBM. Contemporary efforts increasingly leverage the genetic pliability of herpes simplex virus type 1 vectors to insert exogenous payloads and impose temporal control, enabling phase-specific expression of BiTEs and other immunomodulators across the viral life cycle to maximise therapeutic index and antitumour effect 157.

Armed OV platforms are becoming multifunctional. Embedding BiTE expression within viral backbones has emerged as a central strategy in GBM 158. Exploiting OV tropism and intralesional amplification, BiTEs are produced locally and durably within the TME, extending effective half-life while minimising systemic exposure. Following infection, tumour cells synthesise and secrete BiTEs; each infected cell can release thousands of molecules per day, yielding local concentrations approaching ~100 nM—well above those typically achieved with systemic dosing 158. In preclinical models, a single intratumoral administration sustained BiTE expression for more than 60 days, indicating favourable *in vivo* stability 159. The result is multimodal antitumour activity: (i) direct oncolysis; (ii) BiTE-mediated T-cell activation and cytotoxicity; and (iii) TME remodelling with increased CD8⁺ T-cell infiltration and depletion of immunosuppressive populations.

A representative dual-virus paradigm further augments efficacy. Coordinated delivery of an oncolytic adenovirus with a BiTE-expressing adenoviral vector allows the OA to lyse antigen-positive tumour cells and liberate neoantigens, thereby priming local immunity 160, while the EAd infects residual cells to provide sustained intratumoral BiTE expression and T-cell-mediated cytotoxicity. To address distribution and host-immunity barriers, investigators are evaluating convection-enhanced delivery and ultrasound-microbubble-assisted BBB modulation to improve intracerebral spread; genome editing to delete viral immune-evasion genes and mitigate pre-existing immunity; and conditionally replicating constructs to heighten tumour selectivity. This “lysis-plus-delivery” synergy improved outcomes across animal models, with combination cohorts achieving survival rates up to 80%, outperforming single-virus arms 159.

Rational combinations potentiate these effects: pairing OV-BiTE platforms with low-dose chemotherapy, radiotherapy, or immune-checkpoint blockade further enhances efficacy; for example, co-administration with anti-PD-1 increased complete response rates to approximately 75%. From a translational perspective, multiple virus-antibody platforms have progressed to IND-enabling development, some with FDA orphan-drug designation, and phase I dose-escalation trials are assessing safety. Future directions include stimuli-responsive viral systems, real-time imaging for pharmacodynamic monitoring, and individualised regimens tailored to patient immune signatures. Mechanistically, sustained *in situ* BiTE expression circumvents the BBB, elevates local drug exposure, and broadens antigen recognition to counter intratumoral heterogeneity and immune escape. OV-induced oncolysis and BiTE-driven T-cell mobilisation create a positive-feedback loop: release of DAMPs and antigens promotes dendritic-cell maturation and T-cell recruitment, strengthening immune-synapse formation and enabling potent cytotoxicity even when target-antigen expression is low. Ultimately, this strategy suppresses key immunosuppressive mediators (e.g., TGF-β, IL-10), increases infiltration by M1-polarised macrophages and CD8⁺ T cells, and upregulates MHC and costimulatory molecules—collectively establishing a durable antitumour immune milieu.

## DTriTE Design: A Multi-antigen Engager for Overcoming Immune Escape in GBM

To contend with the pronounced heterogeneity of GBM while simultaneously engaging multiple classes of immune effector cells, TsAbs have emerged as a promising next-generation immunotherapeutic modality. By concurrently binding TAAs and receptors on diverse effector populations, TsAbs can mitigate antigen escape and relieve immunosuppression; accordingly, they are increasingly regarded as logical successors to BsAbs in cancer immunotherapy 163.

Most TsAbs adopt a “2+1” architecture, comprising two tumor-antigen-binding domains and one T-cell-engaging module. Engineered linkers promote cooperative epitope recognition and reduce the risk of therapeutic escape 164. In hematologic malignancies, several designs have shown activity: CD3/CD28/CD38 constructs deliver dual T-cell signaling and delay progression in multiple myeloma; CD19/CD22/CD3 or CD19/CD3/CD28 molecules reduce antigen escape in B-cell leukemia 165; and NK-cell-engaging formats such as CD16a/NKp46/TAA counter therapeutic resistance in acute myeloid leukemia 166. In solid tumors, dual-epitope blockade (e.g., EGFR/c-MET) has achieved disease control in advanced lung cancer 167, whereas HER2-directed TsAbs coupled to T- or NK-cell activators (e.g., CD3/CD28 or CD16a/NKG2D) elicit potent immune activation and tumor suppression in breast-cancer models, underscoring the capacity of TsAbs to orchestrate multicellular immune networks 168.

Against this backdrop, GBM—marked by extreme antigenic heterogeneity—serves as a critical testbed for multivalent antibodies. Epidemiologic analyses indicate that EGFRvIII and IL-13Rα2 are expressed in 76.7% and 51.2% of patients, respectively, with co-expression covering >90% of the population. On this basis, Park et al. developed a DNA-encoded dual-tumor-antigen trispecific T-cell engager (DTriTE) to overcome the limited responses associated with single-target BiTE therapy in GBM 169. DTriTE is a single-chain construct integrating scFvs against EGFRvIII, IL-13Rα2, and CD3ε. Flexible linkers optimize domain geometry, enabling stable bridging of two tumor antigens to T cells and enhancing immunological-synapse formation 170. With computer-aided orientation optimization, the molecule improves tumor recognition and cytotoxicity, exhibiting ~150-fold higher apparent affinity for tumor cells than monospecific antibodies and mitigating antigen-loss-driven failure. Functionally, DTriTE induces CD8⁺ T-cell production of IFN-γ and TNF-α, drives CD4⁺ T cells toward cytotoxic differentiation (IL-2, CD107a), and activates NKT cells to release GZMB and perforin—together eliciting a coordinated, multispectrum antitumor response 159, 171.

To address challenges in TsAb development, current strategies prioritize molecular engineering and pharmacologic tuning: Fc fusion to extend half-life; transcytosis-based carriers to enhance BBB penetration; and tumor-microenvironment-restricted, protease-cleavable linkers for conditional activation, thereby improving efficacy while limiting systemic toxicity 172, 173. Structure-function analyses show that spatial placement of the CD3-binding domain critically shapes activity and safety. In the DTriTE variant DT2035, central positioning of the CD3 module preserves antigen targeting while lowering basal T-cell affinity, reducing nonspecific activation and improving synapse balance and efficiency 174. In NSG-K intracranial xenografts modeling GBM heterogeneity, a single DT2035 dose combined with adoptive T-cell transfer maintained effective serum levels for >100 days and significantly improved long-term survival (120-day survival 67% vs ≤33% in controls). Transcriptomics confirmed upregulation of cytotoxic programs (granzyme B, FasL), activation of IL-2R and CDK pathways, and downregulation of immunosuppressive markers (FOXP3, TIGIT), indicating sustained immune activation despite an exhausted milieu 175.

From a translational perspective, multiple TsAb platforms have entered clinical development as regulatory guidance is being articulated. Future directions include stimuli-responsive antibodies, theranostic integrations, and modular plug-and-play architectures, alongside combinations with OVs or immune-checkpoint inhibitors to construct more precise therapeutic networks. Remaining hurdles—molecular complexity, immunogenicity, and manufacturing cost—will necessitate continued cross-disciplinary optimization. Notably, DT2035 retained potent antigen-specific cytotoxicity (56-97%) and multiparametric cytokine secretion (IFN-γ, TNF-α, IL-2) in peripheral blood mononuclear cells from GBM patients even after multiple cycles of chemoradiotherapy, underscoring durability and functional robustness.

In summary, by synergistically targeting EGFRvIII and IL-13Rα2, DTriTE shows promise for addressing GBM's antigenic heterogeneity. Its structural innovations and mechanistic profile provide design principles for future multivalent antibodies and lay a technological foundation for immunotherapy in CNS tumors characterized by complex multi-antigen expression 175.

## Comparative Analysis of GBM Immunotherapies: Mechanisms, Limitations, and Synergies

Immunotherapy for GBM is advancing on multiple fronts. Despite mechanistic diversity, most strategies are constrained by a “cold” TME characterized by sparse TIL, abundant immunosuppressive mediators, and a restrictive BBB. ICIs can activate systemic antitumor immunity and are effective in several malignancies; in GBM, however, phase III testing (e.g., CheckMate 143) has been negative, underscoring the need to increase TIL infiltration and reverse T-cell exhaustion. CAR T-cell and CAR-NK therapies offer antigen specificity and the potential for immunological memory, but face antigen escape, T-cell dysfunction/exhaustion, manufacturing complexity, and risks such as CRS. OVs couple direct oncolysis with immune activation—G47Δ, for example, has shown a survival signal—yet remain vulnerable to clearance by antiviral responses, highlighting the importance of optimising delivery and rational combinations. Vaccine strategies (e.g., DCVax-L) illustrate individualized potential but are limited by TME-mediated suppression and modest response rates. Cytokines (such as IL-2 and IL-15) can rapidly mobilize immunity but are constrained by toxicity and short-lived efficacy.

BsAbs are off-the-shelf agents with intrinsic “bridging” capacity and flexible multi-antigen design; for example, AMG596 has shown early signals of survival benefit. Owing to their modular mechanisms, BsAbs combine readily with ICIs, CAR-based therapies, and OVs, positioning them to address key bottlenecks in GBM immunotherapy.

## Optimizing Clinical Translation: Smart Delivery and Combination Regimens for Durable Responses

Despite steady mechanistic innovation across immunotherapeutic modalities, clinical translation ultimately hinges on solving the complex biology of GBM, and several bottlenecks persist. BsAbs offer a means to surmount traditional limitations via T/NK-cell redirection, coordinated blockade of pro-tumor and immunosuppressive axes (e.g., VEGF/Ang-2; EGFRvIII×CD3), and remodeling of the tumor immune microenvironment (Table 3) 184. Preclinical and early clinical data support their relevance to GBM's core challenges. Representative agents—including the B7-H3×CD3 BsAb MGD009 and the EGFRvIII×CD3 BsAb AMG596—have entered phase I/II testing, providing preliminary signals of CNS tolerability and antitumor activity; the VEGF/Ang-2 BsAb A2V has likewise shown a trend toward survival benefit. These advances reflect the “off-the-shelf” nature of BsAbs, their capacity for precise immune modulation, and progress in delivery (e.g., TfR-targeted approaches).

Nonetheless, fundamental obstacles remain. First, limited BBB penetration and inadequate intracranial exposure constrain efficacy. Although TfR-mediated transcytosis and related strategies can improve CNS delivery, clinical deployment—particularly in the setting of anti-angiogenic-therapy-induced BBB remodeling—must balance molecular size (tissue penetration), half-life, and effector function. Nanoparticle carriers confer targeting advantages in animal models, yet translation is complicated by the human BBB and aberrant vasculature. Systemic dosing often yields subtherapeutic brain concentrations; heterogeneous BBB disruption further limits intratumoral exposure of IgG-like or BiTE formats (e.g., modest enrichment with MGD009), impeding maintenance of therapeutic levels 185.

Second, intratumoral heterogeneity and dynamic antigen plasticity undermine durability. Targets such as EGFRvIII (positive in ~20-30% of cases) display spatial heterogeneity and can be downregulated under therapeutic pressure, enabling outgrowth of antigen-negative clones after initial response. Pathway redundancy (e.g., stemness maintenance via EPHA2/EPHA3) underscores the need for multi-target designs. Although B7-H3×CD3 and EPHA2/EPHA3 BsAbs and TsAbs are effective preclinically, clinical feasibility and off-tumor toxicity require rigorous validation.

Third, a persistently immunosuppressive TME attenuates efficacy. GBM is prototypically “cold”: inadequate T-cell infiltration; enrichment of TAMs and MDSCs (together ~30-50%); accumulation of regulatory T cells; and high TGF-β and IL-10 collectively drive T-cell exhaustion. Potent activation by BsAbs (e.g., BiTE-driven T-cell engagement) also increases the risk of systemic toxicities such as CRS (grade ≥3 with blinatumomab ~15%) and neurotoxicity, necessitating dose modulation, prophylaxis, and Fc-engineering-based risk mitigation. Pharmacokinetics further constrain use: short-half-life BiTEs (e.g., blinatumomab t½ ≈ 2.5 h) require continuous infusion, increasing burden; conversely, long-acting Fc formats may pose chronic safety risks (e.g., hepatotoxicity) when BBB penetration is limited. Intrathecal and other alternative routes are being explored but require stringent procedural safeguards.

Future breakthroughs will likely require coordinated, multidimensional innovation. Technology integration—delivery and protein engineering. Prioritize TfR-targeted systems (e.g., TfR-BsAbs) to enhance intracranial accumulation; deploy microenvironment-responsive carriers (e.g., MMP-2-sensitive nanoparticles) for controlled release; and leverage viral/cellular vehicles (e.g., OV-mediated BiTE gene delivery) for local secretion. Antibody engineering should emphasize affinity/valency tuning (e.g., tetravalent TandAb®), Fc silencing to reduce toxicity, and tumor-activated pro-antibody designs. Develop multispecific constructs (e.g., IL-13Rα2/EGFRvIII×CD3 trispecifics) to address heterogeneity, and consider integrating immune agonists (IL-15 superagonists) or TGF-β traps to potentiate effector function.

Mechanistic synergies with standard and emerging therapies. Combine BsAbs with TMZ/radiotherapy to upregulate targets (e.g., NKG2D ligands) and DAMPs, enhance immune infiltration, and exploit transient radiotherapy-induced BBB permeability to improve penetration; pair with ICIs to reverse T-cell exhaustion; and coordinate with OVs to inflame the TME. In sequential paradigms with CAR-T cells, BsAbs can serve as an off-the-shelf bridge to facilitate trafficking and infiltration. Targeting TAMs/TGF-β can relieve immunosuppression; rational combinations (e.g., an immune-redirecting BsAb plus an anti-angiogenic agent) may yield multi-mechanistic additivity.

Clinical strategy—precision medicine and adaptive trialing. Use multi-omics to select targets (e.g., HER2/EGFR co-expression) for initial therapy; monitor dynamic target loss via circulating tumor DNA and multi-site biopsies; and incorporate molecular subtype (e.g., IDH status) and TME metrics (T-cell clonality, M1:M2 ratios) to refine stratification and endpoints in clinical trials.

In sum, BsAbs open a new therapeutic window in GBM. Realizing their potential will require overcoming delivery bottlenecks, tumor heterogeneity, TME-driven immunosuppression, and toxicity. Through cross-disciplinary integration and rational combinations, BsAb-based strategies could help shift the treatment paradigm toward more durable remission.

## Conclusion

Despite decades of investigation, GBM care remains defined by substantial unmet need. BsAbs—by virtue of multi-mechanistic synergy, off-the-shelf deployability, and high-precision targeting—offer renewed prospects for breaching entrenched therapeutic barriers. This Review synthesizes advances in deploying BsAbs to confront GBM's core obstacles—BBB penetration, intratumoral heterogeneity, and reprogramming of the immunosuppressive TME—spanning innovative target pairings, advanced delivery strategies, and next-generation molecular formats.

Nevertheless, clinical translation remains the rate-limiting step. Principal hurdles include inefficient BBB delivery, dynamic antigen loss with attendant management of heterogeneity, control of immune-related toxicities, and optimization of pharmacokinetics. To maximize the therapeutic potential of BsAbs and move toward durable clinical remission, priorities should include: (1) stimulus-responsive delivery systems to increase intracranial drug exposure; (2) multi-target, cooperative designs to counter antigen escape; (3) antibody-engineering solutions that balance potency with safety (e.g., affinity/valency tuning and Fc modulation); and (4) rational integration with chemotherapy, immune-checkpoint inhibition, oncolytic virotherapy, and cellular therapies to achieve multilayered, mechanism-complementary treatment. Ultimately, deep cross-disciplinary integration—bridging antibody engineering, nanotechnology, cell therapy, virology, tumor immunology, and clinical medicine—will be essential to realize the next generation of “intelligent” BsAbs and rational combination regimens, and to effect a transformative shift in the therapeutic paradigm for GBM.

## Figures and Tables

**Table 1:** Promising Bispecific Antibody Target Combinations for GBM Therapy

Target 1	Target 2	Mechanism of Action	Advantages	Challenge	Development Code	Development Stage	Reference
Ang-2	TSPO	Synergistic blockade (angiogenesis + apoptosis resistance)	Concomitant inhibition of aberrant angiogenesis and anti-apoptotic pathways; Remodeling of the immune microenvironment; Significant extension of survival.	Limited BBB penetration; Residual immunosuppressive TAMs.	ScBsAbs Ang-2/TSPO (Zhou et al.)	Preclinical	45
VEGF	Ang-2	Dual blockade (angiogenesis pathway)	Disrupts VEGF/Ang-2-mediated resistance loop; Promotes vascular normalization; Drives TAM polarization toward M1 phenotype.	Efficacy dependent on vascular phenotype; Restricted BBB delivery.	A2V (CrossMab technology); IBI324	Phase I (NCT01688206)	48
B7-H3	CD3	T-cell redirection	Bypasses MHC-I deficiency; PET imaging shows high tumor enrichment; Synergizes with ferroptosis pathway to enhance efficacy.	Risk of systemic CRS; Iinadequate BBB penetration.	MGD009	Phase I/II (NCT02952259)	64,65
EGFRvIII	CD3	T-cell redirection	Tumor-specific target; Fully humanized design minimizes immunogenicity; Enables potent lysis of heterogeneous tumors.	Risk of antigen loss; Potential for ICANS-related neurotoxicity.	AMG596; BiTE platform	Phase I (NCT03296696; NCT04903795)	69,114
NKG2D	EGFR	NK cell redirection	Overcomes NKG2DL deficiency; IL-15 superagonist boosts NK activity; Addresses antigenic heterogeneity.	Off-tumor risk due to low EGFR expression in normal tissues; NK cell exhaustion; limited BBB access.	NKAB-EGFR (Anne K et al.)	Preclinical	79
NKG2D	CD3	T-cell redirection	Recognizes eight NKG2DLs to overcome ligand heterogeneity; Sensitizes tumors to chemo/radiotherapy; Eliminates GSCs.	Requires chemo/radiotherapy preconditioning; T cell exhaustion concerns.	G207-NKG2D BiTE (Baugh et al.)	Preclinical	89
EPHA2	EPHA3	Synergistic blockade (paracrine signaling)	Targets GSCs stemness maintenance; Induces tumor differentiation; Suppresses AKT/ERK signaling pathways.	Low intracranial delivery efficiency; Target specificity in recurrent tumors.	EPHA2/A3 BsAbs (Sheila K.S et al.)	Preclinical	96
IL-13Rα2	CD3	T-cell redirection	Exhibits high tumor specificity; Induces tissue-resident memory T (T<sub>RM</sub>) cell responses; Resists T cell exhaustion.	Spatial heterogeneity of target expression; Short BiTE half-life.	NSC vector-delivered BiTE (Katarzyna C.P et al.)	Preclinical	99
IL-13Rα2	CD16	NK cell redirection	HLA-independent cytotoxicity; IL-15 enhances NK cell persistence; Reverses immunosuppressive TME.	TGF-β-mediated NK suppression; Efficacy depends on target antigen density.	IL-15-BiTE fusion protein	Preclinical	113

**Table 2:** Comparison of Advanced Brain-Targeted Delivery Strategies for Glioma Bispecific Antibody Therapy

Technology Type	Representative Study	Core Principle	Advantages	Limitations
TfR Targeting 122,140	VEGF-Trap/moAb4 Dual Antibody	Combined TfR targeting to avoid competition Rab4/Rab11 cycling and pathway deactivation Fc mutation (LALAPG) reduces risks	1. BBB penetration remodeling2. Maintenance of TfR membrane status3. Rapid brain parenchymal delivery	Requires optimization of plasma clearance rate Limited clinical data for GBM
Nanocarriers	S-biAb/dEGCG@NPs Platform	MMP-2 cleavable PLGLAG peptide for drug release HA/CD44-targeted BBB penetration Dual antibody + dEGCG co-targeting "immune-escape" & "death" checkpoints	1. Enzyme-controlled drug release2. Immuno- and iron-coordination-mediated3. Deep tumor penetration	MMP-2 overexpression-dependent Complex large-scale manufacturing
Cell Therapy 64,100	NSC-IL13Ra2/CD3 BiTE System	Tumor chemotaxis and migration Continuous BiTE for >7 days Anti-PD-1+/Tim-3+ synergy	1. Long-acting parenchymal delivery2. “Ready-to-use” platform3. Reversible immune escape modulation	Long-term safety evaluation needed Anti-carrier immunity may impact efficacy
Nanovaccines 133	GBM-PDTCM@AuNRs Complex	Patient-derived glioma and immortalized cell fusion SERS multispectral guidance Photothermal therapy synergy	1. Ultra-high BBB/tumor targeting2. Integrated diagnosis and therapy3. Universal drug delivery platform	Complex individualized preparation Requires external light source activation Batch-to-batch variability in carrier membranes
OVs 87,141	G207-NKG2D BiTE	OVs -mediated tumor cell lysis Expresses BiTE for >60 days Dual viral targeting with "avoidance" mechanism	1. Sustained tissue retention (single injection > 60 days)2. Enhanced BBB permeability3. >80% cooperative production yield4. Immune escape microenvironment modulation	Risk of virus-like immunogenicity Route-of-administration limitations (requires intrathecal injection) Manufacturing scalability challenges

**Table 3:** Immunotherapy in the Treatment of GBM: A Comparison

Therapy Type	Mechanism Action	Key Benefits	Limitations	Key Agents	Progress	BsAb Synergy
ICI(PD-1/PD-L1 Inhibitors)^176^, ^177^	Block PD-1/PD-L1 pathway to release T-cell inhibition and enhance anti-tumor immunity.	Systemic immune activation for anti-tumor immunity, prolonged survival (e.g., black color rash)	Low response rate (10-30%) Insufficient T cell infiltration ("cold tumor") Ineffective TME modification	Nivolumab, Pembrolizumab	**Negative**: CheckMate 143 trial (Nivolumab monotherapy OS 10.4 months, no significant survival benefit)	**Cooperative Mechanism**:BsAbs enhance T cell recruitment and reverse immune suppression via ICIs.**Combination Strategy**: BsAb + anti-PD-1 enhances T cell activity.
ICI (CTLA-4 Inhibitors)^178^	Block CTLA-4/B7 interaction to enhance T-cell activation.	Combination with PD-1 inhibitors enhances therapeutic effect (e.g., black color rash)	Limited single-agent efficacy (median OS 4.8 months) High toxicity (3% poor response)	IpilimumabEGFRvIII-CAR-T, IL13Rα2-CAR-T, HER2-CAR-T	**Combination**: Combination with anti-PD-1 (NCT03233152) safe, but limited survival benefit (OS 9.2 months)	**Cooperative Mechanism**:BsAbs activate T cells and inhibit CTLA-4, promoting T cell expansion.**Combination Strategy**: Dual blockade with CTLA-4 inhibition enhances immune response.
CAR-T/NK Therapy^179^	Engineer T/NK cells to target tumor antigens (e.g., EGFRvIII, HER2), directly attacking tumor cells.	High specificity for target cells Persistent anti-tumor memory Potential for blood-brain barrier (BBB) penetration	Immune evasion (e.g., tumor heterogeneity) Ineffective TME modification (e.g., Tregs, MDSCs)	EGFRvIII-CAR-T, IL13Rα2-CAR-T, HER2-CAR-T	**Negative**: NCT02209376 (EGFRvIII-CAR-T infusion, 75% complete remission)	**Cooperative Mechanism**:BsAbs (e.g., BiTE) recruit intrinsic source T cells, but CAR-T numbers are insufficient.**Combination Strategy**: CAR-T + BsAb co-targeting (e.g., EGFRvIII + HER2).
OVs^180^	Selectively infect and lyse tumor cells, releasing tumor antigens and activating systemic immunity.	Direct tumor elimination + immune activation "Origin" vaccines with efficacy in memory response	Antiviral immunity clearance BBB penetration limitations Unstable clinical responses	G47∆ (Japan trial), T-VEC	**Negative**: G47∆ in Japan trials for malignant glioma (II phase trial extended survival)	**Cooperative Mechanism**: OVs releases inflammatory cytokines, enhancing BsAb T cell infiltration.**Combination Strategy**: OV + BsAb (e.g., CCL5-BsAb) enhances T cell activity.
BsAb/BiTE^181^	Combine tumor antigen (e.g., EGFRvIII) and immune cell receptors (CD3) to redirect T-cells to tumor cells.	Precise immune targeting High-efficiency T cell activation (no MHC required) Can target multiple antigens (e.g., EGFRvIII + HER2)	CRS Tumor antigen loss BBB penetration challenges	Blinatumomab, AMG596 (EGFRvIII-BiTE), Mosunetuzumab	**Negative**: NCT05187624 (EGFRvIII-BsAb survival extension)	**Self-Optimization**:Co-targeting with BsAbs (e.g., EGFRvIII + HER2) overcomes tumor resistance.**Combination Strategy**: BsAb in combination with ICI enhances tumor-specific T cell recruitment.
Vaccine Therapy^182^	Target tumor antigens (e.g., EGFRvIII), activate dendritic cells (DCs), and initiate T-cell response	Induces immune memory for targeted antigens Potential for personalized therapy	Ineffective TME modulation Low clinical response (<30%)	DCVax-L, PEP-3-KLH (EGFRvIII vaccine)	**Positive**: The phase III trial of DCVax-L demonstrated extended overall survival (OS), although it did not meet the primary endpoint. **Negative**: Rindopepimut (EGFRvIII vaccine) failed in its phase III trial.	**Cooperative Mechanism**:Vaccination activates T cells, while BsAbs enhance tumor cell destruction.**Combination Strategy**: Vaccination + BsAbs increases T cell expansion efficiency.
Other Cytokines^183^	Administer immune-stimulating cytokines (e.g., IL-2, GM-CSF) to activate immune cells or reverse TME inhibition	Short-term robust anti-tumor immune activity	Systemic toxicity Treatment resistance Difficulty in reversing TME inhibition	IL-2, GM-CSF	**Limited Evidence**: Combination with GD2-BsAb (NCT02173093) safe, but no major therapeutic impact	**Cooperative Mechanism**:Tumor cells support BsAb-induced T cell expansion.**Combination Strategy**: BsAb + IL-2 maintains T cell persistence.

## Abbreviations

BsAbs: bispecific antibodies
BiTEs: bispecific T-cell engagers
scFvs: single-chain variable fragments
ADCC: antibody-dependent cellular cytotoxicity
CDC: complement-dependent cytotoxicity
CRS: cytokine release syndrome
CD3ε: cluster of differentiation 3ε
TAAs: tumor-associated antigens
MHC: major histocompatibility complex
TCR: T-cell receptor
PD-1: programmed cell death protein 1
CTLA-4: cytotoxic T-lymphocyte-associated protein 4
TIM-3: T-cell immunoglobulin and mucin-domain containing-3
GBM: glioblastoma
TME: tumor microenvironment
TsAbs: trispecific antibodies
BBB: blood-brain barrier
TGF-β: transforming growth factor-β
TAMs: tumor-associated macrophages
EGFRvIII: epidermal growth factor receptor vIII
IL-13Rα2: interleukin-13 receptor subunit alpha-2
HER2: human epidermal growth factor receptor 2
Ang-2: angiopoietin-2
TSPO: translocator protein
VEGF: vascular endothelial growth factor
B7-H3: B7 homolog 3
SPR: surface plasmon resonance
IFN-γ: interferon-γ
IL-2: interleukin-2
MMP-2: matrix metalloproteinase-2
HA: hyaluronic acid
GPX4: glutathione peroxidase 4
PET: positron-emission tomography
TRAEs: treatment-related adverse events
NKG2D: natural killer group 2D
IL-15: interleukin-15
GZMB: granzyme B
TMZ: temozolomide
GSCs: glioma stem cells
OVs: oncolytic virus
EPHA2: ephrin type-A receptor 2
EPHA3: ephrin type-A receptor 3
rGBM: recurrent glioblastoma
TfR: transferrin receptor
CNS: central nervous system
RMT: receptor-mediated transcytosis
FDA: US Food and Drug Administration
TIL: tumor-infiltrating lymphocyte
DAMPs: damage-associated molecular patterns
AuNRs: gold nanorods
GBM-PDTCM@AuNRs: theranostic nanocomplex
SERS: surface-enhanced Raman scattering
DTriTE: dual-tumor-antigen trispecific T-cell engager
